# Avian cryptochrome 4 binds superoxide

**DOI:** 10.1016/j.csbj.2023.12.009

**Published:** 2023-12-18

**Authors:** Jean Deviers, Fabien Cailliez, Aurélien de la Lande, Daniel R. Kattnig

**Affiliations:** a Living Systems Institute and Department of Physics, University of Exeter, Stocker Road, Exeter, Devon, EX4 4QD, United Kingdom; b Institut de Chimie Physique, CNRS UMR 8000, Université Paris-Saclay, 91405 Orsay, France

## Abstract

Flavin-binding cryptochromes are blue-light sensitive photoreceptors that have been implicated with magnetoreception in some species. The photocycle involves an intra-protein photo-reduction of the flavin cofactor, generating a magnetosensitive radical pair, and its subsequent re-oxidation. Superoxide (O${}_{2}^{•-}$) is generated in the re-oxidation with molecular oxygen. The resulting O${}_{2}^{•-}$-containing radical pairs have also been hypothesised to underpin various magnetosensitive traits, but due to fast spin relaxation when tumbling in solution would require immobilisation. We here describe our insights in the binding of superoxide to cryptochrome 4 from *C. livia* based on extensive all-atom molecular dynamics studies and density-functional theory calculations. The positively charged “crypt” region that leads to the flavin binding pocket transiently binds O${}_{2}^{•-}$ at 5 flexible binding sites centred on arginine residues. Typical binding times amounted to tens of nanoseconds, but exceptional binding events extended to several hundreds of nanoseconds and slowed the rotational diffusion, thereby realising rotational correlation times as large as 1 ns. The binding sites are particularly efficient in scavenging superoxide escaping from a putative generation site close to the flavin-cofactor, possibly implying a functional relevance. We discuss our findings in view of a potential magnetosensitivity of biological flavin semiquinone/superoxide radical pairs.

## Graphical abstract

**Figure:**
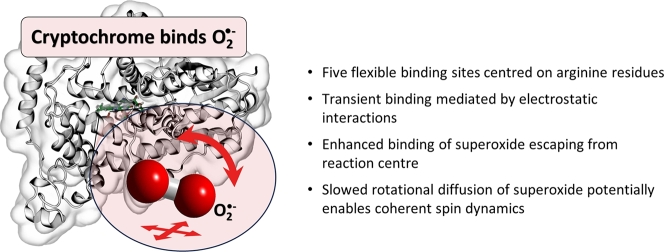

## 1 Introduction

**Fig. 1:**
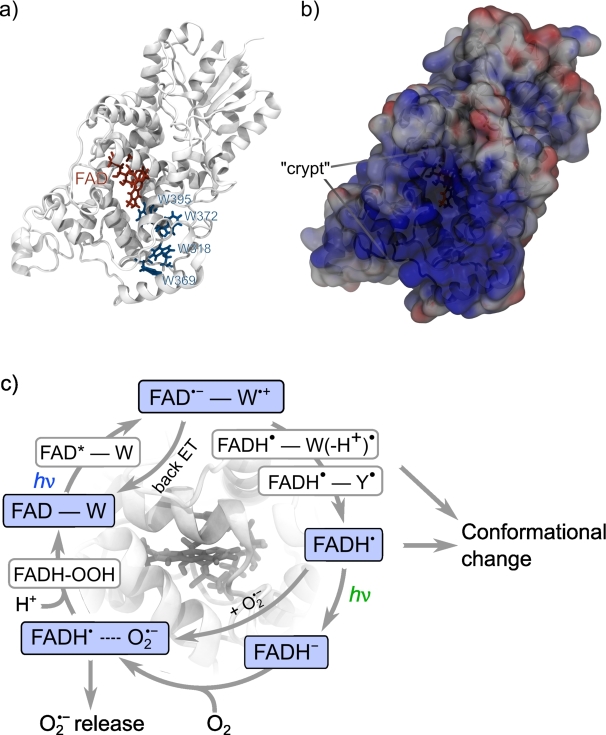
Representation of the *Cl*Cry4 protein and the redox-cycle underpinning magnetoreception. a) Illustration of the protein secondary structure highlighting the FAD cofactor and the tryptophan tetrad. b) Electrostatic potential at the protein surface, whereby blue and red denote positive and negative potential, respectively. The protein orientation is identical to that of a), providing a scenic view of the “crypt”, i.e. the cavity leading to the flavin binding region. A central pore leads to the FAD and the putative superoxide reaction site studied here (cf. Fig. 6b). c) Putative cyclic reaction scheme of *Cl*Cry4 comprising the photo-reduction of the resting state (binding fully oxidised FAD) to the semi-quinone (binding FADH^•^) and finally the fully reduced form (binding FADH^−^), the re-oxidation with molecular oxygen generates superoxide, O${}_{2}^{•-}$. Magnetosensitivity can result at the branching points involving radical pairs. This is established for FAD^•−^/W^•+^ and debated for FADH^•^/O${}_{2}^{•-}$. In the latter case, binding of the O${}_{2}^{•-}$ is essential to counter fast spin relaxation.

For a range of organisms, most notably night-migratory songbirds such as the European robin, specific magnetosensitive traits, such as a compass sense, are attributed to quantum spin dynamics in radical pairs [1], [2], [3], [4]. The sensory protein is thought to be the flavoprotein cryptochrome (Fig. 1), which acquires magnetosensitivity through photoexcitation. However, there are many remaining open questions [5]. In the canonical model (Fig. 1c, upper branch), absorption of short-wavelength photons by the protein-bound co-factor FAD initiates a cascade of electron transfer processes along a chain of tryptophan residues–the tryptophan tetrad–that leads to the formation of a spin-correlated radical pair between the cofactor and surface exposed tryptophan(s) [6]. The spin-state of this radical pair evolves between electronic singlet and triplet states as a consequence of magnetic interactions, which modulates its ability to undergo spin-selective back electron transfer to reform the resting state [1]. Ultimately, this imparts magnetosensitivity on the yield of follow-up products that result from the radical pair in spin-independent reactions, such as protonation reactions or protein structural rearrangements. These stabilised states, which still comprise the flavin co-factor in its reduced, semi-quinoic form, are thought to link to a signalling cascade *via*, as of now, unknown interaction partners [6]. The reaction cycle is eventually closed, i.e. the cryptochrome resting state is regenerated, on a much slower timescale, by re-oxidation, possibly following an additional photo-reduction step that generates the fully reduced FAD cofactor (Fig. 1c, lower branch) [7]. The most obvious oxidant is molecular oxygen, for which the re-oxidation generates superoxide radicals, O${}_{2}^{•-}$, amongst various reactive oxygen species (ROS), at least transiently [8]. The induced cellular redox changes could also have potential signalling roles [5], [9].

The flavin reoxidation step itself has been implicated with magnetosensitivity although this proposition remains controversial [8], [10]. Specifically, a transient flavin semiquinone-superoxide radical pair has been considered to underpin cryptochrome's magnetic sensitivity. This hypothesis dates to the early days of the field, spurred by the observation of resonance-like behaviour in the radio-frequency magnetic field sensitivity of birds' ability to orient, which suggested the involvement of a radical devoid of hyperfine interactions, such as superoxide [11], [12]. More recently, this hypothesis has been revived by observations of cryptochrome-associated magneto-receptive traits in birds and plants in the dark, which appear to preclude a directly light-dependent pathway such as photo-reduction in favour of dark-state reoxidation [13], [14], [15]. The contentious suggestion of magnetosensitive superoxide-radical pairs has faced opposition due to superoxide's electronic structure (Π122), which implies strong spin-orbit coupling, accompanied by swift spin relaxation through the spin rotational mechanism, which entirely suppress radical pair-based magnetosensitivity in weak magnetic fields if the molecule is freely tumbling in solution [10], [16], [17]. This criticism is often countered by the argument that the superoxide could be immobilised in cryptochrome, sufficiently lengthening its rotational correlation time such that magnetosensitivity can be sustained. However, so far no study seems to have addressed the nature and effectivity of the envisaged immobilisation with reference to realistic protein-superoxide interactions. Alternatively, three-radical processes involving a scavenging reaction can acquire magnetosensitivity even in the limit of instantaneous spin relaxation in superoxide [18], [19], [20]. However, these models are complex and not yet directly supported by experimental studies. As superoxide-containing, putatively magnetosensitive radical pairs have now been suggested in diverse biological contexts, the question of its magnetosensitivity, promoted via binding, extends beyond magnetoreception [21], [22], [23], [20].

More broadly, the superoxide radical anion [24], O${}_{2}^{•-}$, is involved in various metabolic processes within the cell, such as respiration through the electron transfer flavoprotein (ETF) complexes [25], phagocytosis [26], or phosphorylation-induced signalling [27] to initiate processes such as mitosis, cell differentiation, or apoptosis, whereby a baseline level is required for normal cell function [28], [29]. O${}_{2}^{•-}$ radicals are typically formed in flavo- or quinoenzymes through a one-electron transfer from a reduced flavin or quinone donor to an oxygen molecule [30], as is the case for cryptochrome [8]. While superoxide generation and release can occasionally be the desired outcome (e.g. as a phagocytising agent released from NADPH oxidase [31]), it often manifests as a toxic phenomenon that needs to be controlled (e.g. O${}_{2}^{•-}$ leaking from the ETF chain and presumably cryptochrome reoxidation). Indeed, although a moderately oxidative ion, high concentrations of O${}_{2}^{•-}$ in cells are a source of oxidative stress [32], either through its own action or through conversion into more potent oxidants, such as the hydroxyl radical [24]. As such, it is thought to participate in the cell-degrading process of aging. Chronically elevated oxidant levels have furthermore been linked to the onset of various pathologies [33]. In this context, the question of whether cryptochrome is able to guide and trap superoxide at the reaction site is central, and, stipulated by its one-electron transfer redox cycle, can be posed irrespective of the identity of the actual magnetosensitive radical pair. The ability of proteins to sequester small molecules for extended (e.g. tens of nanoseconds) lengths of time is well documented [34]. Arginine, lysine and, to a lesser extent, glutamine have been implicated with the electrostatic guidance of O${}_{2}^{•-}$ to the metal centre of an antioxidant enzyme [35], [36], [37], [38]. However, whether cryptochrome has comparable properties is unknown.

Here, we report our efforts to address this question through extensive all-atom molecular dynamics simulations of the cryptochrome 4 protein from *Columba livia*, *Cl*Cry4. We address four scenarios aimed at revealing the protein's ability to sequester, bind and immobilise superoxide. Firstly, we have identified binding sites through simulations involving super-physiological concentrations of bulk superoxide. Secondly, we have evaluated the binding characteristics in terms of binding times and increases in the anion's rotational correlation time for individual superoxide anions initiated at these binding sites. Thirdly, we report on the ability of said binding sites to sequester superoxide generated at a possible solvent-accessible superoxide formation site in the vicinity of the FAD. Finally, we describe superoxide's dynamics adjacent to the FAD inside a solvent-inaccessible, closed pocket. We discuss our findings in terms of the cryptochrome sensing cycle and the putative magnetosensitivity attributed to the re-oxidation of the flavin cofactor, which is a recurring, widely suggested, but controversially discussed pattern of biological magnetosensitivity for cryptochrome and beyond.

## 2 Methods

MD simulations were carried out using the GPU-accelerated “pmemd.cuda” code in AMBER 18 [39], [40], [41]. The *Cl*Cry4 protein was modelled using the ff14SB forcefield [42]; the reparametrised version of the AMBER Generalised All-Atom Force-Field (GAFF2) was employed for the semiquinone flavin adenine dinucleotide semiquinone FADH^•^ and the superoxide O${}_{2}^{•-}$ [39]. The former used RESP atomic charges derived in [43] and the latter symmetrically distributed partial charges of −0.5 and the experimentally determined equilibrium bond length. TIP3P water was employed to solvate the system in combination with GAFF2 Cl^−^ and Na^+^ ions.

System heating and equilibration was carried out following a protocol outlined in [44], where a series of equilibrations in the NpT (target pressure: 1.0 bar, using a Berendsen barostat [45]) and NVT thermodynamical ensembles allowed to prepare a solvated protein system at the desired pressure and temperature. In these and all following MD simulations, temperature control was achieved with the use of Langevin dynamics, using a collision frequency of $\gamma_{\ln}=2.0\;{\mathrm{ps}}^{-1}$ [46].

A total of four distinct sets of MD simulations were created. All production runs were generated in the NVT ensemble, at a temperature of 313.0 K, employing periodic boundary conditions. Electrostatic interactions were computed with the Particle Mesh Ewald (PME) summation method, with a cutoff for short-range at 9.0 Å [47]. A 2.0 fs integration timestep was used throughout, and hydrogen bonds were constrained using the SHAKE algorithm [48].

The first set of MD simulations (later referred to as **I**) were carried out on a 89637-atom system of solvated *Cl*Cry4-FADH^•^ complex in a 87×89×117 Å^3^ periodic box, with 21 O${}_{2}^{•-}$. 10 replicas, differing by the initial positions of the superoxide radicals (randomly located using packmol [49]), were created and, after equilibration, ran for 500ns each, saving geometries every 200ps. The second set of simulations (**II**) used a 89617-atom system, taken from 22 distinct frames from set **I** in which 20 of the 21 O${}_{2}^{•-}$ were mutated into Cl^−^. For each of the starting configurations, 20 replicas were generated, resulting in a total of 440 MD runs with geometries saved every 10ps. The third set (**III**) of MD simulations consisted of 100 replicas of a frame initially extracted from set **II**, where the superoxide ion was moved (using a VMD script) to a putative formation site near the flavin. Geometries for the resulting 100 MD runs were saved to trajectory every 200ps. Finally, the fourth set of MD simulations was carried out for a 90563-atom system of solvated *Cl*Cry4-FADH^•^ complex in a 99×86×114 Å^3^ periodic box, with 17 Na^+^, 19 Cl^−^, and a single O${}_{2}^{•-}$ radical located in a cavity near the flavin. A total of 1μs of production runs were accumulated, saving geometries every 10ps.

Single-point energy calculations were carried out using Gaussian 16 [50]. O${}_{2}^{•-}$/Arg pairs' interaction energies were computed by DFT at the CAM-B3LYP [51]/def2-TZVP [52] level with D3BJ correction of dispersion forces [53]. The Basis Set Superposition Error (BSSE) was eliminated using the Counterpoise method [54], [55]. The desolvation energy of O${}_{2}^{•-}$ and Cl^−^ ions of various explicit water clusters, modelled in Molden [56], were computed using DFT at the CAM-B3LYP/def2-TZVPP level, with GD3BJ correction for dispersion. Implicit solvation was included using the IEF-PCM model [57].

Superoxide *g*-tensors were calculated for 17 configurations of the anion bound in the FAD-binding pocket (extracted from fourth set of MD simulations) using DFT in Orca [58]. The B3LYP functional was used in combination with the def2-TZVP basis set and D3BJ dispersion correction [53]. The environment was represented through point charges; effective core potentials were added for point charges closer than 3Å to the superoxide to prevent overpolarisation of the electron density.

## 3 Results

### 3.1 Bulk superoxide binding to *Cl*Cry4 (**I**)

We have undertaken extensive all-atom molecular dynamics simulation of cryptochrome 4 from *Columba livia*, *Cl*Cry4, binding the FAD cofactor in its semiquinone form, FADH^•^. The starting configuration corresponded to the most frequently occurring conformation as observed in our previous study, based on a clustering analysis of the trajectory of a 800-ns MD simulation [59]. The starting point of this earlier study was the crystal structure of *Cl*Cry4, as deposited in the PDB (identifier: 6PU0), with the phosphate binding loop reconstructed as described in [60]. The structure was solvated and equilibrated (in the NpT ensemble for T=313 K and p=1 bar) as described in detail in the Methods section. Production simulations were run for the NVT ensemble. Simulations were undertaken using superoxide anions or chloride anions as the counterions to sodium cations and the four-fold positively charged protein (17 Na^+^ ions and 21 anions in an equilibrated volume of 87×89×117 Å^3^; anion concentration 38 mM). In total, 10 replication runs of 500 ns each, sampled every 200 ps, were generated for both anions with the aim to identify anion-protein interaction sites and motifs. A Root-Mean-Square Deviation (RMSD) analysis of the positions of the protein backbone relative to the starting configuration was in line with the expectation for a well-equilibrated *Cl*Cry4 system and amounted to 1.70±0.65 Å and 1.93±0.98 Å for the combined trajectories containing O${}_{2}^{•-}$ and Cl^−^, respectively. Refer to Fig. S1 in the SI for individual RMSD trajectories. Root-Mean Square Fluctuations (RMSF) of atomic positions, i.e. the average RMSD value for individual residues with respect to the average structure, identify the phosphate-binding loop (PBL; residues 228 to 244) as the most flexible section of the backbone for both sets of trajectories (see Fig. S2 in SI). In addition, the two adjacent segments roughly comprising residues 180-195 and 200-210 showed marked mobility (see Fig. S3 in the SI for a protein structure with highlighted mobile segments). These observations are in line with a previous study by Schuhmann et al. that identified the PBL in *Cl*Cry4 as a versatile region in simulations of the FAD^•−^/W${}_{D}^{•+}$ radical pair [60]. As shown in Figs. S4 and S5 in the SI, occasional jumps in the RMSD as a function of time typically correlated with fluctuations in the identified mobile segments.

**Fig. 2:**
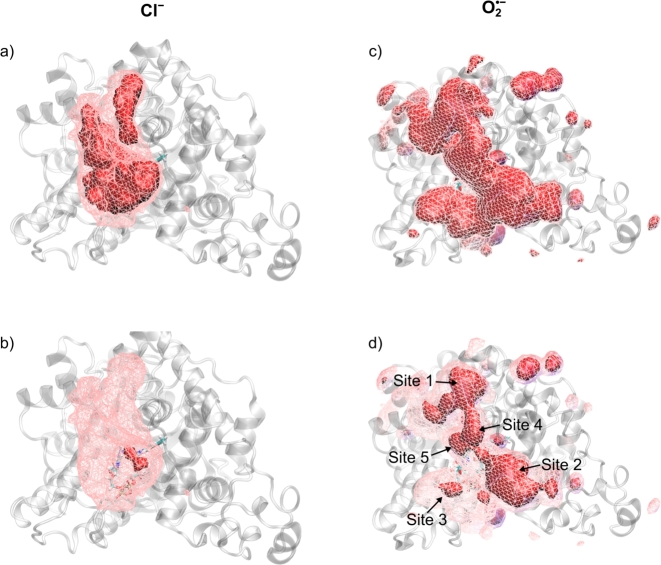
Volumetric density maps of Cl^−^ (panel a and b) and O${}_{2}^{•-}$ (c and d) ions, computed using the VMD plugin VolMap over the 10 500-ns trajectories, concatenated and aligned onto the first frame of the Cl^−^-containing trajectory for best visual comparability. For all representations, transparent wireframe meshes materialise an isodensity of 0.0060 Å^3^; red solid surfaces, an isodensity of 0.0066 Å^3^ (a and c) or 0.015 Å^3^ (b and d). For d, strongly binding regions in vicinity of the FAD have been labelled.

We have aligned the combined trajectories comprising a total of 5 μs for each anion to a common reference (the first frame of a Cl^−^-containing trajectory) and used the VolMap tool in VMD [61] to calculate a three-dimensional histogram of anion occupancy. Fig. 2 provides iso-density plots comparing the occupancy for O${}_{2}^{•-}$ and Cl^−^. The voxels size was set to 1×1×1 Å^3^ and two contour levels, 0.0066 and 0.015, were chosen. A contour level of 0.015 implies that every cubic bin within the iso-surface has an associated probability of 1.5 percents or higher to contain an ion, at any given frame. Fig. 2 reveals surprising differences between O${}_{2}^{•-}$ and Cl^−^. While both anions prefer locations in the “crypt” of *Cl*Cry4, i.e. the concave region of the protein shape leading to the flavin binding cavity (see Fig. 1b), the O${}_{2}^{•-}$ residency is more spread out at the 0.0066 contour level and shows marked hotspots within the “crypt” in close vicinity to the protein surface at the 0.015 level. This distribution into localised sites suggests a particular affinity with specific residues. On the contrary, for Cl^−^, only a single, comparably remote hotspot is seen on the 0.015 level, suggesting a reduced tendency to localisation relative to O${}_{2}^{•-}$. The overall preference of both anions for the “crypt” area is in agreement with the electrostatic landscape of the protein, i.e. the surface electrostatic potential, which is markedly positive in this region, as shown in Fig. 1b. For O${}_{2}^{•-}$, the structured hotspot distribution motivates the analysis in terms of five distinct sites, which have been indicated in Fig. 2d. Table 1 identifies these sites in terms of the superoxide-binding residues (details discussed below).

**Table 1:** Amino acids involved in the binding of O${}_{2}^{•-}$ for the binding sites identified in Fig. 2d.

Site	Residues
1	Arg497, Arg486, Arg490
2	Arg415, Arg419
3	Thr209, Arg217, Arg218
4	Arg409
5	Lys234, Arg236

**Fig. 3:**
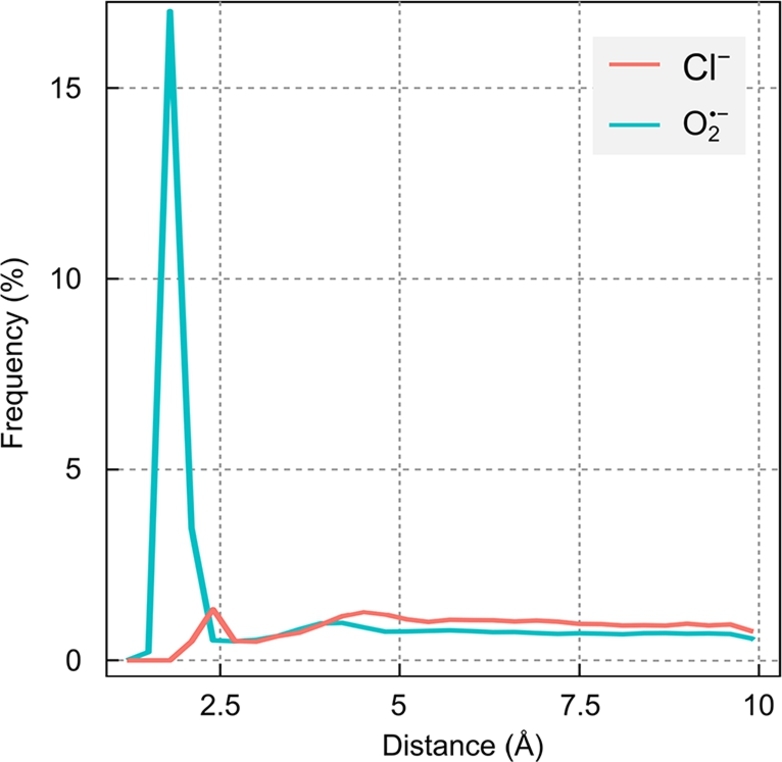
Density plot of the shortest distances between a protein heavy-atom and O${}_{2}^{•-}$ (blue) and Cl^−^ (red), calculated over 10 500-ns MD simulations (10 × 2500 frames) and averaged over 21 ions for each set. For O${}_{2}^{•-}$, the ion-protein distance is measured with respect to whichever oxygen atom is closest.

Fig. 3 shows plots of the probability density of the shortest distances between a protein atom and an anion. The representation highlights a much larger probability of O${}_{2}^{•-}$ to reside in direct contact with the protein than is the case for Cl^−^. Integrating the first peak for both ions, an O${}_{2}^{•-}$ ion has a 21% probability of being found within 2.75 Å of the protein surface, while Cl^−^ has a 3% probability of being within 3.00 Å. The average distances corresponding to the peaks amount to 1.87 Å and 2.45 Å for O${}_{2}^{•-}$ and Cl^−^, respectively. For both ions, a second minor peak occurs at larger distances from the protein surface, which corresponds to the ions solvated with one shell of water molecules. For both ions, this constitutes a minor binding modality (solvation is further explored in the Discussion).

Fig. S6 in the SI shows the probability that an ion-binding event involves a specific amino acid residue as the nearest neighbour to the ion. Here, a binding event has been defined as a configuration with protein-anion distance below 4 Å for at least 2 consecutive molecular dynamics frames (sampling interval: 200 ps). For O${}_{2}^{•-}$, over the 5 μs combined trajectories 121,235 such encounters were recorded; for Cl^−^ only 3879 events met this criterion (out of a total of 525,000, i.e. 2,500 frames × 10 trajectories × 21 ions). The residue resolved statistic reveals an overwhelming preference of O${}_{2}^{•-}$ for arginine (Arg; 40% of binding events), followed by lysine (Lys; 7%) and leucine (Leu; 5%). For Cl^−^, arginine is also the most relevant binding partner, but it only accounts for 21% of the events. The affinity of anions for Arg and Lys is readily explained by their positive charge and abundance in the crypt region. On the other hand, the relatively large tendency for binding to the neutral Leu is surprising at first glance. Visual inspection of the crypt region suggests that its relatively large abundance here is likely related to the presence of Leu residues next to strongly binding residues. Indeed, Leu314 is located adjacent to Arg486, one of the key amino acids for immobilising O${}_{2}^{•-}$; Leu354 is between Arg409 and Arg419, two flexible residues which have been observed to exchange O${}_{2}^{•-}$ radicals (see Fig. S7 in SI).

**Fig. 4:**
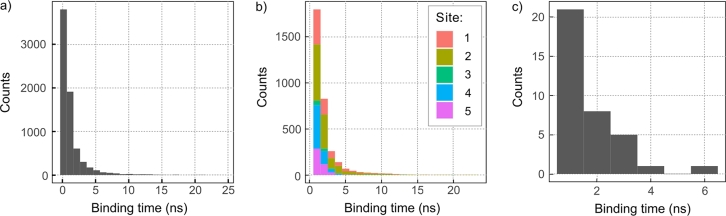
Histogram of the lengths of O${}_{2}^{•-}$ (a and b) and Cl^−^-protein binding events (c). Binding events are defined as encounters of at least 2 consecutive frames (spanning 0.4 ns) for which a given ion is located within 4 Å of a protein heavy-atom. For b) and c) we only consider binding event spanning at least 1 ns. In b), we consider binding events at binding sites as defined in Table 1 and visualised in Fig. 2d. For O${}_{2}^{•-}$, the binding time histograms have been truncated; a small number of long-lived binding interactions is not shown (cf. Table 2).

We have further analysed the length of binding interactions. Fig. 4 reports the distribution of binding times $t_{b}$. Binding interactions shorter than 0.4 ns (2 consecutive frames) have been omitted. For superoxide, the graph was further truncated at $t_{b}=25$ ns and therefore does not show a low density of long bindings. Focusing on the O${}_{2}^{•-}$ results, we note that most binding events are shorter than 5 ns, but, crucially, longer binding is possible: we witnessed 133 binding events with $t_{b}\geq 10$ ns, and 2 with $t_{b}\geq 100$ ns; the longest event spanned 148.6 ns. Note that most binding events are associated with the 5 auspicious sites identified above. Table 2 classifies the binding events at the 5 binding sites by their binding time. Out of a total of 10,203 recorded encounters, 32.3% trapped O${}_{2}^{•-}$ for longer than 1 ns. Site 2 is the most effective at binding O${}_{2}^{•-}$ from the solution (31.0% of all binding events), closely followed by site 4 (26.0%). Site 3 has the smallest number of binding events (only 3.0%), but is the only site that shows binding longer than 100 ns. The average binding time over all sites, which is the maximum likelihood estimator of the inverse decay rate assuming an exponential distribution of binding times, amounts to 1.1 ns. Fitting the time-dependence of the bound fraction, i.e. the fraction of initially bound configurations that are still bound at a particular time [62], using a bi-exponential function reveals a dominant binding modality with time constant 0.93 ns besides a minor component with time constant 15 ns and 1.1% fractional contribution (Fig. S8). For binding site 3, the average binding time is markedly longer (2.4 ns) and the minor component grows in size (1.8%) and markedly in the characteristic unbinding time (117 ns). The characteristic unbinding times are summarised for all binding sites in Table S1 in the SI. Note however that, for reasons that will become apparent later, we view the summary of binding times, as presented in Table 2, as more indicative. A closer analysis of binding patterns shows that the binding typically involves a single, solvent-exposed arginine. Double-binding occasionally occurs at site 2, i.e. R415 + R419, and, to a much lesser extent, cross-site for R409 + R415 (see Fig. S7 in the SI for details). Except for Arg490 and Arg217, which undergo H-bonding with adjacent Glu residues, the involved Arg-residues are free, i.e. not integrated in a stable intra-protein hydrogen bonding network at their positively charged guanidino-groups.

**Table 2:** Number of binding events (BE) observed for different conditions, including I) the 10 500-ns trajectories of 21 freely-diffusing O${}_{2}^{•-}$; II) 440 restarted trajectories tracking the escape of a single O${}_{2}^{•-}$ from a binding site; and III) 100 trajectories of a single O${}_{2}^{•-}$ diffusing from a putative formation site near the flavin.

Condition	Site	Total # of BE	# with $t_{b}\geq 1\;\mathrm{ns}$	# with $t_{b}\geq 10\;\mathrm{ns}$	# with $t_{b}\geq 100\;\mathrm{ns}$
I	1	2113	764 (36.2%)	26 (1.2%)	0
	2	3165	1339 (42.3%)	44 (1.4%)	0
	3	304	94 (30.9%)	5 (1.6%)	2 (0.7%)
	4	2660	670 (25.2%)	2 (0.1%)	0
	5	1961	432 (22.0%)	0	0

II	1	100	67 (67.0%)	11 (11.0%)	0
	2	100	66 (66.0%)	18 (18.0%)	0
	3	100	88 (88.0%)	58 (58.0%)	16 (16.0%)
	4	40	21 (52.5%)	1 (2.5%)	0
	5	100	6 (6.0%)	0	0

III	1	15	12 (80.0%)	1 (6.7%)	0
	2	164	134 (81.7%)	22 (13.4%)	1 (0.6%)
	3	0	0	0	0
	4	107	79 (73.8%)	7 (6.5%)	1 (0.9%)
	5	212	149 (70.3%)	15 (7.1%)	0

### 3.2 Superoxide escaping from binding sites (**II**)

To characterise the binding interactions of superoxide at the auspicious sites in more detail and preclude collective effects of the super-physiological O${}_{2}^{•-}$ concentration used to identify binding sites, we have generated a separate set of MD trajectories. A single O${}_{2}^{•-}$ was placed in the site of interest and its escape trajectory monitored. Simulations were terminated when the ion separated by more than 3 Å from the residues defining the sites (cf. Table 1). For each site except site 4, five starting geometries were extracted from the exploratory simulations from above; for site 4, only 2 configurations were studied due to its perceived lesser relevance as a bridge connecting sites 1 and 2. The initial configurations were all chosen from a long binding event, to increase the chance of them corresponding to a strongly binding configuration. Each starting configuration was copied into 20 identical replicas, for a total of 100 trajectories for each site (except site 4, with 40).

**Fig. 5:**
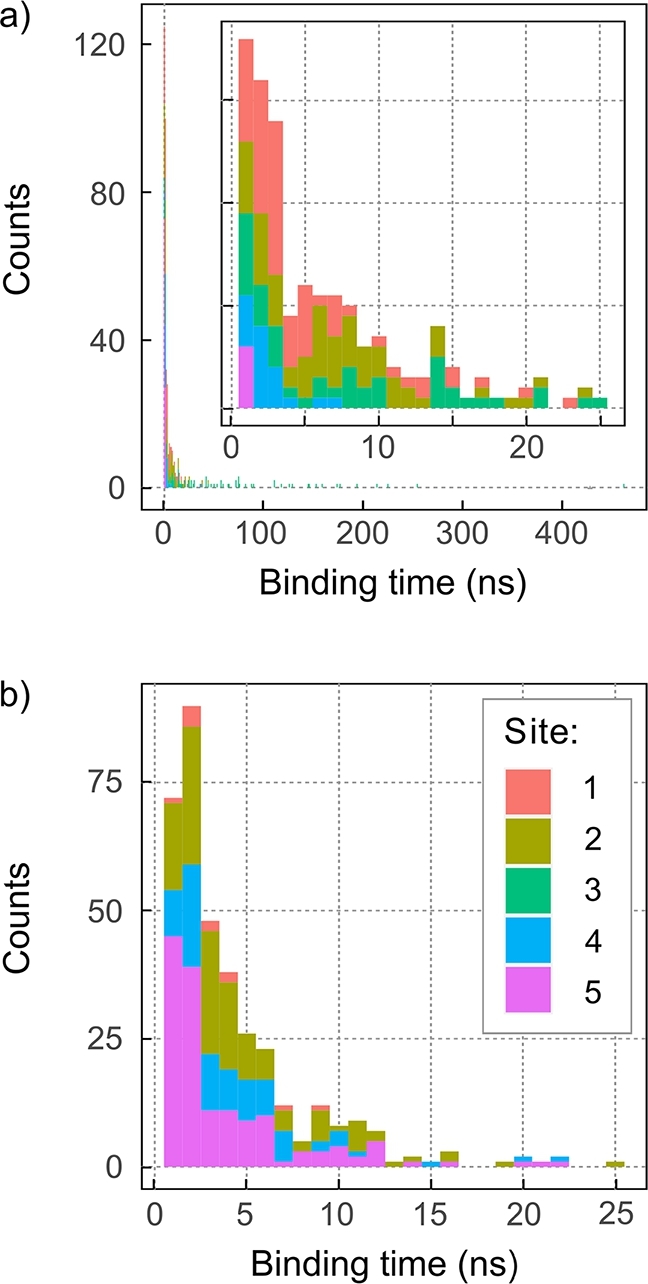
Histogram of the lengths of O${}_{2}^{•-}$-protein binding events for a) the escape of the anion from the binding sites and b) the escape from the putative formation site shown in Fig. 6b. For each initial configuration, the binding statistics are based on 20 MD trajectories, each recorded for 5 initial binding configurations, i.e. a total of 100 trajectories per starting configuration. The detailed histograms comprise binding events longer than 1 ns and leave out rare long-time binding. For a) the outer figure shows a zoomed-out histogram including rare events and binding events with *t*_*b*_ > 400 ps.

Table 2 and Fig. 5a summarise the binding time distribution for these escape simulations. The “best” retention of O${}_{2}^{•-}$ at its starting site was observed for site 3, which yielded binding times of more than 100 ns in 16% and 10 ns in 58% of the simulations. The longest binding time observed amounted to $t_{b}=463.07$ ns. On the other extreme, site 5 yielded $t_{b}>1$ ns in only 6% of the simulations. In general, we observe a strong dependence on the starting configuration (details summarised in SI; Tables S2 - S6), which prevents us from interpreting our results here to reflect well-defined and statistically robust properties of individual sites. Rather, the reader is advised to view the results as an exploration of the question of whether extended binding and immobilisation are achievable in principle. In this respect, our choice of starting configurations seems to have been rewarding for site 3, but failure to observe long-time binding cannot be interpreted as a lack of binding potential at a specific site. Averaging over all binding times nonetheless, we find a mean binding time of 13.5 ns. A bi-exponential fit of the bound fraction, as above [62], yields the time constants 1.90 ns and 52 ns, the latter with a relative weight of 21.2%. For the best binding site 3, the corresponding times reach 5.0 ns and 75 ns, the latter accounting for as much as 62.3% of the events. Please refer to the SI (Table S1) for details.

We have determined the rotational correlation times, $\tau_{2}$, associated with bound configurations. $\tau_{2}$, defined as the characteristic decay time of the auto-correlation function of $\frac{1}{2}(3\cos^{2}(\theta (t))-1)$, where θ(t) is the angle subtended by the O${}_{2}^{•-}$ bond vector, and this vector at time 0, determines superoxide's spin relaxation rates. Again, in view of the diversity of binding propensities observed over the ensemble of studied configurations, we focus on the best, i.e. longest, rotational correlation times, which would give rise to the slowest spin relaxation rates through the spin-rotational mechanism. Table 3 collects the top-10 $\tau_{2}$ values, with their associated $t_{b}$ and spin relaxation rates based on the spin rotational mechanism; averages for sites and individual starting configurations are provided in the SI. For free O${}_{2}^{•-}$, a separate set of simulations with high time resolution yielded $\tau_{2}=0.99\pm 0.33$ ps. The best-performing immobilisation sites are sites 1 to 3, which routinely achieve rotational correlation times on the order of 100 ps. The very best immobilisation, although probably a rare event, is achieved by site 2 and corresponds to a $\tau_{2}$ of almost 1 ns, i.e. a slow-down by a factor of 1000 relative to free superoxide. An intriguing aspect of long-$\tau_{2}$ trajectories is that they do not necessarily exhibit the longest $t_{b}$. Observe, for example, that out of the 10 trajectories with the longest $\tau_{2}$, 4 are shorter than the average binding time, $\langle t_{b}\rangle =23.36$ ns, over the set of trajectories, excluding those that did not lead to immobilisation, as assessed by the criterion $\tau_{2}>10$ ps. A detailed analysis summarised in the SI reveals the absence of correlation of $t_{b}$ and $\tau_{2}$ in terms of the Pearson correlation coefficient. Furthermore, while hydrogen-bonding appears to contribute to the binding times, the rotational dynamics appear uncorrelated with the degree of H-bonding with the arginine residues (Fig. S12). Overall, the ensemble of “bound” configurations is structurally versatile and characterised by retained, but slowed rotational mobility, and intermittent hydrogen bonding at the guanidino-group involving one or both of the *ε*-nitrogen atoms as H-donors (Fig. S11 in the SI illustrates selected bound configurations).

**Table 3:** Top-10 *τ*_2_-values for simulations involving the escape of a single O${}_{2}^{•-}$ from a binding site, reported together with their binding time, *t*_*b*_, and spin relaxation time due to the spin-rotional mechanism, *T* (see Discussion for details on spin relaxation).

Site	*τ*_2_ (ps)	*t*_*b*_ (ns)	*T* (ns)
2	941	44.4	730
1	757	13.3	590
2	455	8.4	350
1	445	11.2	350
3	413	27.3	320
3	401	174.2	310
3	399	118.2	310
3	391	41.9	300
3	374	75.3	290
1	335	15.3	260

### 3.3 Superoxide escaping from a putative reaction site (**III**)

One potential pathway for superoxide creation is the re-oxidation of the flavin cofactor with molecular oxygen. This implies the generation of the anion in the vicinity of the co-factor via electron transfer. To assess whether a O${}_{2}^{•-}$ generated in this way is likely to engage in binding interactions, we have studied the escape of superoxide from putative generation sites.

**Fig. 6:**
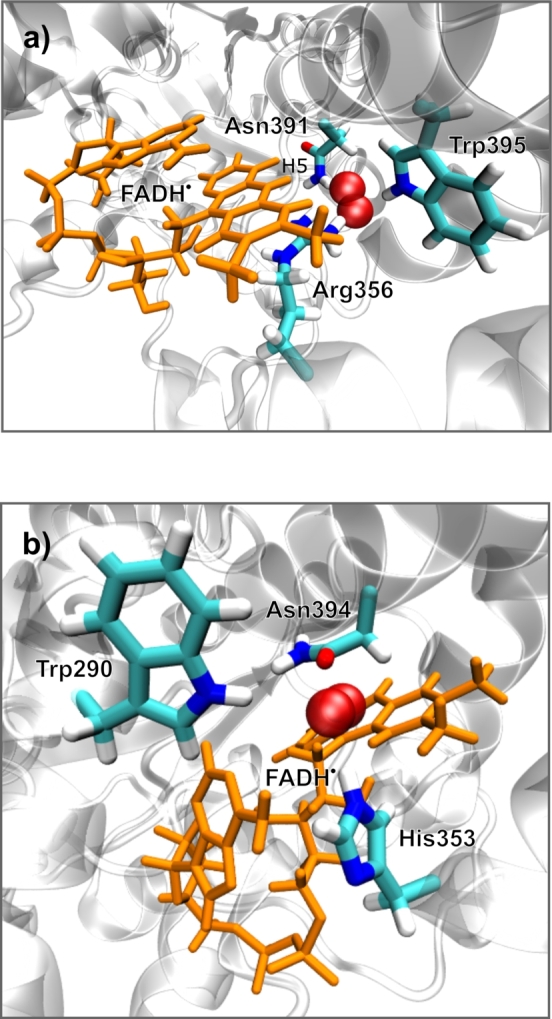
O${}_{2}^{•-}$ in bound configurations close the FAD cofactor. a) illustrates a configuration with O${}_{2}^{•-}$ contained in the FAD-binding cavity resembling Ref. 63. b) shows O${}_{2}^{•-}$ at a putative reaction site accessible from the bulk and in close vicinity of the FAD. The FAD cofactor is shown in orange; protein residues in close contact with the O${}_{2}^{•-}$ have been highlighted.

Mondal and co-workers have previously described superoxide bound to the cryptochrome from *D. melanogaster*, generated by the explicit placement of superoxide into the FAD binding pocket [63]. We have replicated this configuration for *Cl*Cry4, leading to a protein/superoxide complex with O${}_{2}^{•-}$ snugly bound in the binding pocket, directly facing H5 of FADH^•^ and surrounded by Asn391, Trp395 and Arg356 (see Fig. 6a). This configuration did not exhibit a tendency of dissociation over 800 ns of molecular dynamics trajectory (NVT, T=313 K), i.e. the superoxide remained tightly bound throughout. In fact, this configuration also appeared to be inaccessible from the bulk, as it was not populated in any of the molecular dynamics simulations with bulk superoxide (5 μs of production). These observations raise the question of whether this configuration is truly functional.

In view of the superoxide escape of the described superoxide/*Cl*Cry4 complex being beyond the timescale accessible in our simulations, we have studied a second, more reachable configuration. We realised that superoxide could readily approach the FAD-cofactor to 3.5 Å from the C8 methyl group, the point of closest approach putting O${}_{2}^{•-}$ in between His353, Asn394 and Trp290 (see Fig. 6b). To assess the escape dynamics of O${}_{2}^{•-}$ from this putative generation site, we have generated 5 starting configurations (by running a 40 ns MD simulation with O${}_{2}^{•-}$ held at the starting site through a harmonic constraining potential and extracting snapshots after 2, 10, 20, 30 and 40 ns, respectively) and ran 20 replicative MD simulations each. The runs were terminated when the O${}_{2}^{•-}$ strayed away by more than 10 Å from any of the 5 superoxide binding sites identified above.

From the 100 MD simulations generated, 71 led to subsequent binding events at the 5 binding sites. The average simulation length was $\langle t_{s}\rangle =41.11\pm 53.69$ ns, with $t_{s}$ ranging from 3.80 to 294.40 ns (total simulation time: 3,074.8 ns). During this time, O${}_{2}^{•-}$ spent on average 30.33 ns (74% of $\langle t_{s}\rangle$) bound to any of the 5 binding sites (as assessed by the distance being smaller than 3 Å), and the remainder circulating between sites. In total, 498 individual binding events were recorded. Fig. 5 (panel b) provides a histogram of the associated binding times and Table 2 provides a numerical breakdown (Condition III in the Table). The number of binding events over the simulation time equate to a sizeable hit rate of 0.16 events/ns, which markedly exceeds the binding rate of the free-diffusion study (0.09 events/ns). It is also noteworthy that for the escape trajectories, the chance that an encounter leads to extended binding exceeds that of the bulk-initiated simulations. For example, averaged over all sites, 75.1% of encounters led to binding exceeding 1 ns, whereas the odds only amounted to only 32.3% for free diffusion (cf. Table 2). This trend persists for $t_{b}\geq 10$ ns, with an overall 9.0% of all binding events falling into this range. The most efficient binder is site 2, followed by site 1, which bind more than 80% of the encountered O${}_{2}^{•-}$. The most binding events are registered by site 5 (42.6% of the total). Site 3, which was already difficult to populate from the bulk, registers no binding events. The average binding time for all sites amounts to 4.7 ns. However, a bi-exponential analysis of the bound fraction as a function of time, reveals a marked contribution of long binders with time constant ≃50 ns for sites 1, 2, and 4 (see the SI for details). Finally, the largest change in prevalence with respect to the free diffusion simulations is seen for site 1, the contribution of which drops from 31.0% to 3.0%.

## 4 Discussion

We have used extensive all-atom molecular dynamics simulations to infer *Cl*Cry4's propensity to bind the superoxide anion. The data revealed intermittent binding of up to several hundreds of nanoseconds, which appears to be essentially driven by the Coulomb interaction. On the mesoscopic scale, the positively charged “crypt” region is the preferred interaction site; on the microscopic level, five binding sites have been identified, the binding interactions at which are mainly mediated by surface exposed arginine residues. The binding times at these sites varied strongly with the initial configuration. In exceptionable cases, binding, defined as lasting close contact, over hundreds of nanoseconds was observed. Binding did not immobilise the superoxide, which retained a degree of rotational mobility, but was slowed compared to that in free solution. Under the most favourable conditions, the rotational correlation time approached 1 ns, a 1000-fold increase over free rotational diffusion.

The asymmetric and aggregated appearance of superoxide binding sites at a protein face permitting access to the flavin binding site raises speculations about its functional relevance. Meanwhile, the notion of the electrostatic guidance of a reactive species to a reaction site is a well-documented phenomenon, notably for reactive oxygen species [35], [36], [64], [38], [37]. We envisage that the crypt of the *Cl*Cry4 acts as a funnel holding O${}_{2}^{•-}$ within electron-transfer range of the flavin, thereby both favouring its efficient recombination, and, importantly, reducing the efflux of ROS, thus mitigating the potential cellular toxicity associated with cryptochrome re-oxidation. This notion is corroborated by the observation that superoxide radicals escaping from the putative formation site are captured more efficiently than bulk superoxide. Potentially, binding could also enable magnetosensitivity of a superoxide-containing radical pair or radical triad, which will be discussed below.

We here have probed the interaction of the protein with superoxides escaping from a putative reaction site, scavenged from bulk solutions, and bound in the FAD cavity. Except for the latter, it is unlikely that the identified binding sites coincide with the generation sites (involving the re-oxidation of the flavin cofactor with molecular oxygen) or constitute the sites of follow-up reaction closing the reaction cycle (involving generation of hydrogen peroxide). However, superoxide binding is still expected to impact on the overall efficiency of the re-oxidation by preventing its escape into the bulk. In this context, a recent study by Salerno et al. is noteworthy, as it explores the binding of molecular oxygen to a plant cryptochrome [62]. While this study identified several well-defined binding sites, oxygen bound in these sites was found essentially unreactive towards the reduced FAD cofactor, as the binding of oxygen in an apolar environment rendered the electron transfer endergonic. This suggests that superoxide and molecular oxygen binding sites are disparate and that the reoxidation likely involves the fleeting diffusive encounter of the reactants rather than pre-binding of oxygen. This also indirectly motivates the approach used here to identify binding sites starting from free or escaping superoxide, rather than bound molecular oxygen.

The force fields employed in molecular dynamics simulations are in general not parametrised to replicate amino acid anion binding interactions and care must be exercised not to overinterpret specific findings. With this caveat in mind, we have validated the forcefield in terms of its ability to reproduce binding energies of the two relevant anions, Cl^−^ and O${}_{2}^{•-}$, at arginine sidechains. To this end, 36 isolated arginine-superoxide complexes, excised from representative MD runs, were studied with respect to the intermolecular non-bonded interaction energy (defined in eq. (S1) in the SI) on the molecular mechanics and DFT (CAM-B3LYP/def2-TZVP with D3BJ dispersion correction) level of theory. To study the Arg-Cl^−^ interaction, the O${}_{2}^{•-}$ in the complexes was mutated to Cl^−^ and its position re-optimised, before proceeding as above. Detailed results are provided in the SI (Table S7). The bottom line of this analysis is that the force field underestimates the interaction energy for both anions by about 20%; specifically, −21.2 kcal/mol (19%) for O${}_{2}^{•-}$, and −22.8 kcal/mol (21%) for Cl^−^. Based on this, we can conclude that, crucially, the O${}_{2}^{•-}$ interaction energies are not overestimated by the forcefield, meaning that the length and quality of immobilisation of binding events as reported here likely constitute a lower bound, and that rotational correlation times computed herein could in fact be longer. It will be important to extend the study of Arg-O${}_{2}^{•-}$ binding in the future, e.g. by using QM/MM or purpose-parametrised forcefields.

**Fig. 7:**
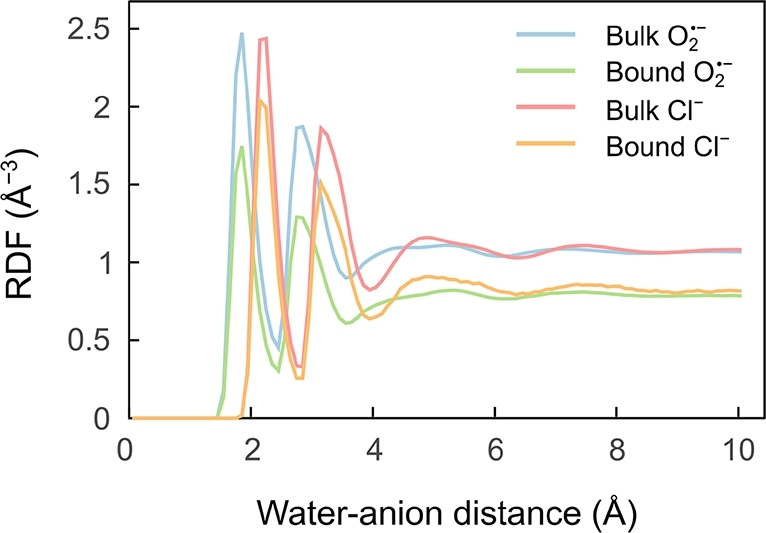
Radial Distribution Functions (RDF) of water around O${}_{2}^{•-}$ and Cl^−^ when in close contact with the protein (ion-protein distance smaller than 3.0 Å) or bulk (distance from protein larger than 10.0 Å). The RDFs have been computed over all frames from the 10 500-ns MD simulations, and all ions that satisfied the distance criteria (out of a total of 21).

The observations from above suggest that the binding of O${}_{2}^{•-}$ to Arg is energetically favoured over Cl^−^ binding, at least for the plain ions. Likewise, electrostatic toy models predict smaller electrostatic potential energy of O${}_{2}^{•-}$ and an idealised guanidino group for perpendicular alignment, as a consequence of the smaller ion radius (see Fig. S11 in the SI). In the actual encounter events, however, the situation is obscured by the ions being at least partly solvated by water molecules. A different degree of solvation of the anions is expected to be maintained for ions bound to the protein and in the bulk. Due to the stabilising nature of solvation, the removal of water molecules upon binding at the protein surface comes with an energy penalty, the free energy of desolvation, the relevance of which we aim to discern now. Fig. 7 reports the radial distribution functions (RDF; explicit expression provided in eq. (S2) in the SI) of water around O${}_{2}^{•-}$ and Cl^−^, distinguishing ions in the bound (d<3 Å) and bulk state as extracted from the bulk-diffusion MD study described above (5 μs, 21 anions). It is apparent that both ions exhibit a far-ranging solvation structure with at least 4 readily identifiable solvation shells, both for the bulk and the surface-bound state. For O${}_{2}^{•-}$, the solvation shells peak at shorter distances from the anion than for Cl^−^; this is consistent with the observation in Fig. 7 that O${}_{2}^{•-}$ tends to approach the protein surface more closely. Integrating the RDF yields the number of water molecules as a function of distance from the anions (Fig. S9 in the SI), and can therefore be used to count the number of water molecules in a given solvation shell. The results are summarised in Table 4. On average, Cl^−^ is solvated by more water molecules in the 1^st^ shell than O${}_{2}^{•-}$. Upon binding, O${}_{2}^{•-}$ sheds on average 1.6 water molecules while Cl^−^ loses 1.3. In the SI, we describe our approach for estimating the desolvation free energy (defined in eq. (S3) in the SI) associated with these transitions based on the solvation energies of water-anion complexes with 1 to 6 water molecules (Fig. S10), evaluated using DFT (CAMB3LYP/def2-TZVPP level of theory with GD3BJ empirical dispersion) in combination with a polarisable continuum model (PCM). For the degrees of 1^st^-shell solvation, as outlined in Table 4 for the bulk and protein-bound states, our approach predicts a desolvation free energy of 70 kcal/mol and 72 kcal/mol for O${}_{2}^{•-}$ and Cl^−^, respectively. These estimations are incomplete insofar as they neglect entropic effects in the first solvation shell and the use of a plausible, but arbitrary, octahedral solvent arrangement. Yet, assuming that any such effects are comparable for both ions, the close similarity in desolvation energies suggests that desolvation is not a strong driver, if at all, in the binding capabilities of each ion to the protein. We can therefore make the proposition that the strong differences between binding affinities of O${}_{2}^{•-}$ and Cl^−^ are related to specific ion-side chain interaction energies, rather than desolvation. Still, desolvation could be the decisive factor in explaining the larger capture efficiency of superoxide escaping from the protein pore relative to bulk superoxide. In the former case, the escaping superoxide is partially desolvated from the start and remains so as it diffuses along the protein surface, while in the latter case the need of desolvation manifests through decreased binding efficiency.

**Table 4:** Solvation shell boundary distances, *r*_shell_, and number of water molecules, *n*, within the first two shells as obtained from the integrated RDFs of water around O${}_{2}^{•-}$ and Cl^−^, when the ions are in a bound (*d* ≤ 3.0 Å) or bulk (*d* ≥ 10.0 Å) configuration. The RDFs have been computed for all frames of the 10 500-ns MD simulations, and for all ions (out of 21) satisfying the distance criteria.

	$1^{\text{st}}$ shell			$2^{\text{nd}}$ shell		
	*r*_shell_ (Å)	*n*_bulk_	*n*_bound_	*r*_shell_ (Å)	*n*_bulk_	*n*_bound_
O${}_{2}^{•-}$	2.45	5.22	3.63	3.55	20.57	14.17
Cl^−^	2.85	6.92	5.61	3.95	25.71	20.62

The putative magnetic field sensitivity of FADH^•^/O${}_{2}^{•-}$ and other superoxide-containing radical pairs is a recurring motif when it comes to biological magnetosensitivity and magnetoreception [21], [22], [23], [20]. The proposition is attractive insofar as it could provide magnetosensitivity in processes that are not light-induced, at least not directly, and as the lack of hyperfine-coupled nuclear spins in O${}_{2}^{•-}$ earns it the predicate of a reference-probe system [65], [66]. Such systems, which involve all hyperfine coupled nuclei coupled to one radical partner and none to the other, are renowned for their exquisite sensitivity in weak magnetic fields, such as the geomagnetic field, as its spin dynamics would be controlled by the unperturbed precession of the isolated electron spin in the applied field. However, the sensitivity of radical pairs cannot be assessed independent of their dynamic properties, which unfortunately exposes superoxide's sinister side. Specifically, the orbitally degenerate ground-state electronic structure of O${}_{2}^{•-}$ implicates strong spin-orbit coupling, and thus a strong coupling of spin motion to the anion's molecular tumbling motion, which gives rise to swift spin relaxation via the spin rotational mechanism [67], [16], [17]. Assuming isotropic rotational diffusion, the spin relaxation rate, $T^{-1}$, is given by

(1) $$T^{-1}=\frac{1}{9}\frac{\Delta g^{2}}{\tau_{2}},$$

where $\tau_{2}$ is the rotational correlation time and $\Delta g^{2}=\sum_{i=1}^{3}{\left(g_{ii}-g_{e}\right)}^{2}$, with $g_{ii}$ denoting the principal values of the *g*-interaction matrix and $g_{e}$ the free-electron *g*-factor [17]. For a rotational correlation time of 1 ps, as for free O${}_{2}^{•-}$, and a modest $\Delta g^{2}=0.0116$ (which reflects a significant quenching of the orbital angular momentum via the crystal field splitting; $g_{11}=2.0020$, $g_{22}=2.0077$, $g_{33}=2.1100$; DFT-based QM/MM-prediction for 17 representative MD snapshots of the cavity-bound superoxide using B3LYP/def2-TZVP with D3BJ dispersion correction in Orca [58]), eq. (1) predicts T=0.8 ns. This evidently compares unfavourably with the timescale $\tau_{s}={\left(|\gamma |B/(2\pi )\right)}^{-1}$ of spin precession in weak applied magnetic fields *B*. In the geomagnetic field (50 μT), $\tau_{s}=710$ ns. For $\tau_{s}\gg T$, the spin-correlation in the radical pair is lost prior to spin dynamics acquiring magnetosensitivity and no magnetic field sensitivity is detectable. Indeed, superoxide-containing radical pairs have so far only been found to show magnetic field effects in ultra-high magnetic fields, where the Δ*g*-mechanism permits the acceleration of the singlet-triplet interconversion to timescales faster than *T* [17].

The obvious, widely acknowledged, approaches to overcome this impasse are superoxide binding or non-specific immobilisation, which increase the rotational correlation time and potentially reduce $\Delta g^{2}$ by quenching the orbital angular momentum [67]. For this reason, the evaluation of superoxide-protein binding is central to assessing whether many, arguably bold, suggestions on superoxide-related magnetic field effects are feasible or even plausible. This is pertinent to the question of dark-state magnetoreception in cryptochrome [13], [14], [15], but extends to other quantum biological propositions, such as bioenergetics [21], hypomagnetic field effects on neurogenesis [20], [68], Xe-anaesthesia [69], therapeutic effects of magnetic field exposure [70], etc. (see Ref. 23 for a review).

We have found that binding times of hundreds of nanoseconds and rotational correlation times of up to 1 ns appear feasible in principle. While far off from optimality for magnetoreception, if taken together these parameters promise sizeable magnetosensitivity even in weak magnetic fields, not least because of the comparably huge intrinsic magnetosensitivity that results from the reference-probe configuration in the absence of spin relaxation [65], [71]. Note furthermore that the predicted rotational correlation times would still be fast relative to spin evolution, suggesting that no non-averaged additional hyperfine interactions with the surroundings would become apparent, unlike previously dreaded [67].

While the most extreme observed $t_{b}$ and $\tau_{2}$ allow at least to not reject magnetosensitivity, it is apparent that such promising long binding events manifested only sporadically and strongly depended on the initial configuration. The majority of binding interactions are in fact too short-lived to appreciably impact the spin relaxation rates, and a long $t_{b}$ is not necessarily accompanied by slow rotational dynamics. While this sheds some doubts on the functional relevance of binding, it might be sufficient to explain a low level of background magnetic field sensitivity. For a dedicated sensing mechanism, on the other hand, one would probably expect a more robust binding interaction that efficiently scavenges O${}_{2}^{•-}$ regardless of the initial configuration. Whether this situation could be better realised in the (more viscous) cellular environment is, however, a question that this study cannot assess beyond the point of providing evidence of principal feasibility in the isolated protein. An optimised sensing system would also call for a more direct interrelation of generation, scavenging and follow-on reaction sites, which is not obvious for the 5 identified binding sites. Further studies of the entire re-oxidation processes, starting from molecular oxygen and finishing with the release of H_2_O_2_, will be expedient.

Finally, we note that superoxide bound in the FAD cavity facing N5 (Fig. 6a) tumbles even slower than the slowest rotators identified for the five binding sites (effective correlation time $\tau_{2}=2.9$ (ns)). However, even though this site promises slower spin rotational relaxation, magnetosensitivity can only ensue if the superoxide can escape from the cavity on the timescale of the coherence time, which was not observed in our simulations. Specifically, magnetosensitivity rests on a balance of spin-selective recombination and other pathways, such as escape – a single reaction channel is insufficient. This implies that, for FADH^•^/O${}_{2}^{•-}$ to develop magnetosensitivity, the extent of binding must be well balanced in order to slow spin relaxation while still permitting escape reactions. Based on our observations of 10−100 ns bindings, an increase of the (micro-)viscosity by a factor of approximately 100 to 10 is predicted to establish the best sensitivity, which suggests that surface-exposed positions resembling our sites might be susceptible in cellular environments. However, effects will necessarily be strongly attenuated relative to those calculated for idealised, relaxation-free systems in the literature due to the need to compromise opposing requirements, such as slowing relaxation while permitting bifurcating reaction pathways. Furthermore, suppressive effects of inter-radical interactions, in particular the electron-electron dipolar interaction, of bound superoxide are then unavoidable [72]. In this regard, three-radical effects [73] or the quantum Zeno effect [74] can offer a partial resolution. Studies of more complex reaction/diffusion scenarios will be necessary to conclude on the question of putative low-field magnetosensitivity of superoxide-containing radical systems in the biological context.

## 5 Conclusions

We have studied the transient binding of superoxide to cryptochrome 4 from *C. livia* using all-atom molecular dynamics. The positively charged “crypt” region that leads to the FAD binding pocket transiently binds O${}_{2}^{•-}$. Five localised binding sites centred around arginine residues have been identified within feasible electron transfer distances from the FAD. Typical binding times of the order of 10 ns were found for the aqueous system at 313 K. Exceptional binding events extended to several hundreds of nanoseconds. The binding affinity of superoxide markedly exceeds that of the chloride ion, which only binds sporadically with binding times shorter by at least one order of magnitude and without showing marked localisation, however the “crypt” is still the preferred interaction region. These differences appear to be the consequence of interaction potential energies and unrelated to differences in desolvation free energies. The capture and binding efficiency of superoxide at the identified binding sites strongly depends on the initial configuration of the superoxide-protein complex. Compared to binding from the bulk solution, binding is particularly efficient for superoxide escaping from a putative formation site adjacent to the FAD co-factor, likely because of the favourable spatial correlation between the generation and binding sites and dispensing of desolvation. This could suggest a functional role of the binding sites and positive-potential region to prevent the escape of toxic superoxide from the FAD binding region upon cryptochrome reoxidation, following its photo-reduction through intra-protein electron transfer along the tryptophan tetrad. The superoxide binding is often associated with an increase in the rotational correlation time (although binding time and rotational correlation time do not appear to be correlated). We have evidenced an up to 1000-fold slowdown of the rotational tumbling upon binding of superoxide relative to its rotational diffusion rate in free solution. This implies that spin relaxation rates due to superoxide's spin rotational interaction would be significantly reduced relative to free superoxide, approaching relaxation times on the order of 100 ns. This is sufficient to instigate magnetic field effects from the flavin-semiquinone/superoxide radical pair, at least in moderate magnetic fields. While this supports the idea of magnetosensitivity in the dark-state re-oxidation, we stress that this only applies to the most favourable binding events, which only occurred rarely in our simulations and were strongly dependent on the initial configuration. This suggests that the processes could possibly underpin a bottom-line magnetosensitivity, but, at least based on the current observations, is unlikely to form part of a specialised magnetoreception system, for which efficiency and, as such, a higher degree of orchestration appear necessary. However, one must also not forget that the cellular environment is more viscous and highly crowded. Binding interactions that appear fleeting in our simulations might gain efficiency in the cellular context. In this context, it is noteworthy to point out that the expected increase of the dynamic viscosity associated with the cellular environment would place the binding dynamics in a region suitable for the detection of weak magnetic fields comparable to the Earth's. Additional studies will be necessary to identify the superoxide formation site (through MD focusing on O_2_ binding), the nature of the superoxide-protein interaction (through QM/MM studies and improved forcefields), and the spin dynamics of radical pairs, and radical triads, involving bound superoxide (including a detailed treatment of spin relaxation in view of repeated binding-unbinding events). More broadly, the specific role of arginine in superoxide binding deserves wider investigation, beyond cryptochrome.

## Declaration of Competing Interest

The authors declare that they have no known competing financial interests or personal relationships that could have appeared to influence the work reported in this paper.

## Data Availability

The data that support the findings of this study are available from the authors upon reasonable request.

## Appendix A Supplementary material

MMC The supplementary material file ‘ClCry_binds_superoxide_SI.pdf’ provides additional data and analysis on protein structural fluctuations, the affinity of anions to various amino acid residues, superoxide binding sites, unbinding times, detailed statistic for superoxide escaping from binding site, a comparison of binding energies of anions to arginine as derived from molecular mechanics and DFT, calculations of free energies of desolvation, the role of H-bonding, and a toy electrostatic model. MMC
