# Supplementary material for: Avian cryptochrome 4 binds superoxide
Source: Comput Struct Biotechnol J. 2023 Dec 18;26:11–21. doi: 10.1016/j.csbj.2023.12.009 (PMC10776438; doi:10.1016/j.csbj.2023.12.009)
Supplement: MMC — The supplementary material file ‘ClCry_binds_superoxide_SI.pdf’ provides additional data and analysis on protein structural fluctuations, the affinity of anions to various amino acid residues, superoxide binding sites, unbinding times, detailed statistic for superoxide escaping from binding site, a comparison of binding energies of anions to arginine as derived from molecular mechanics and DFT, calculations of free energies of desolvation, the role of H-bonding, and a toy electrostatic model. [file mmc1.pdf]

Avian cryptochrome 4 binds superoxide
Supporting Information

Jean Deviers,[1,2] Fabien Cailliez,[2] Aurélien de la Lande,[2] and Daniel R. Kattnig[1,*]

[1]*Living Systems Institute and Department of Physics, University of Exeter, Stocker Road, Exeter, Devon, EX4 4QD, United Kingdom*

[2]*Institut de Chimie Physique, CNRS UMR 8000, Université Paris-Saclay, 91405 Orsay, France*

(Dated: December 6, 2023)

PROTEIN STRUCTURAL FLUCTUATIONS

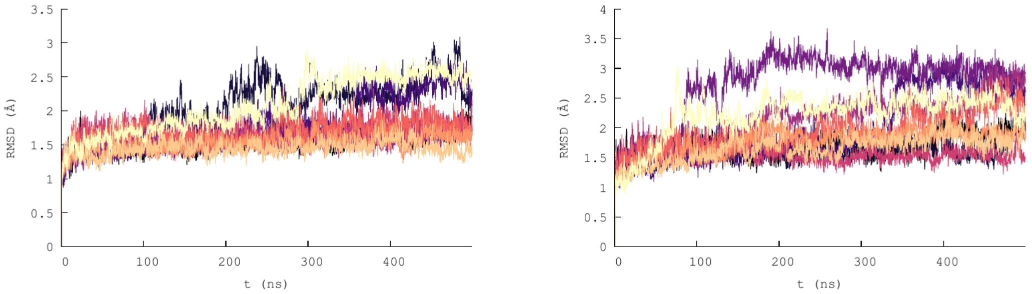

(a) $O_2^{\bullet -}$ (b) Cl^-

Figure S1: Evolution of the Root-Mean-Square Deviation (RMSD) of the positions of protein backbone heavy atoms (`AMBER` names: N, CA, C, O) over ten 500-ns trajectories of solvated *Cl*Cry4 containing FADH$^\bullet$ and 21 (a) $O_2^{\bullet -}$ or (b) Cl^- ions. The majority of trajectories are characterised by a stable RMSD of $\sim 1.5 - 2$ Å, suggesting a conserved secondary and tertiary structure of the protein. Some trajectories exhibit a jump, followed by an elevated but stable RMSD. This is typically associated with the rearrangement of the phosphate binding loop (residues 228 - 244) and two mobile sites (residues 180 - 195 and 200 - 210), in agreement with a previous study [1]. On average, the RMSD amounts to 1.70 ± 0.65 Å and 1.93 ± 0.98 Å for the $O_2^{\bullet -}$ and Cl^- simulations.

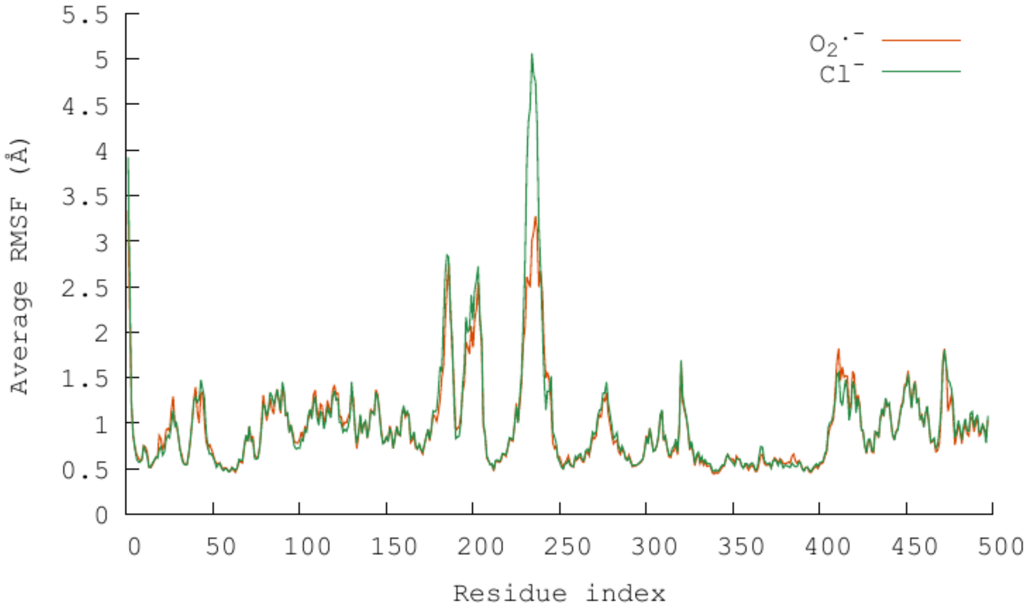

(a)

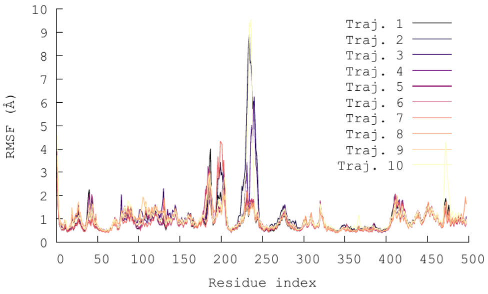

(b) $O_2^{\bullet -}$

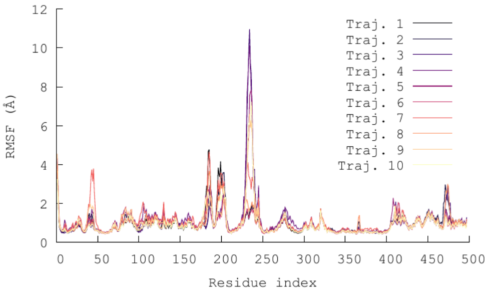

(c) Cl^-

Figure S2: Root-Mean Square Fluctuations (RMSF; i.e. the average RMSD value for a given residue along the trajectory) of the positions of protein backbone heavy atoms, for ten 500-ns trajectories of solvated *Cl*Cry4 binding FADH$^\bullet$ using 21 (b) $O_2^{\bullet -}$ or (c) Cl^- as counterions. (a) reports the average RMSF curve of both data sets. This residue-disaggregated view allows to identify the most mobile parts of the protein. The most flexible sections of the backbone are identified as the phosphate-binding loop (residues 228 to 244), a result confirmed in a previous study [1], and two adjacent regions roughly covering residues 180 - 195 and residues 200 - 210. The RMSF-data show some variability in the magnitude of peaks depending on the simulation, but their location remains consistent across both data sets.

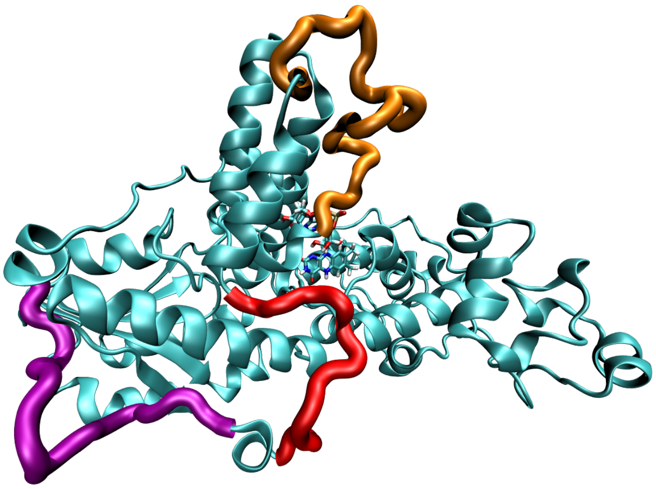

Figure S3: Graphical representation of the starting conformation of the *Cl*Cry4 protein, as derived from the 6PU0 crystall structure. The three most flexible regions are highlighted: residues 228 to 244 (phosphate-binding loop) in gold, 180 - 195 in purple, 200 - 210 in red. These three mobile regions correspond to unstructured, solvent-exposed loops. A well-structured 5-residue alpha helix separates the purple and the red mobile segments.

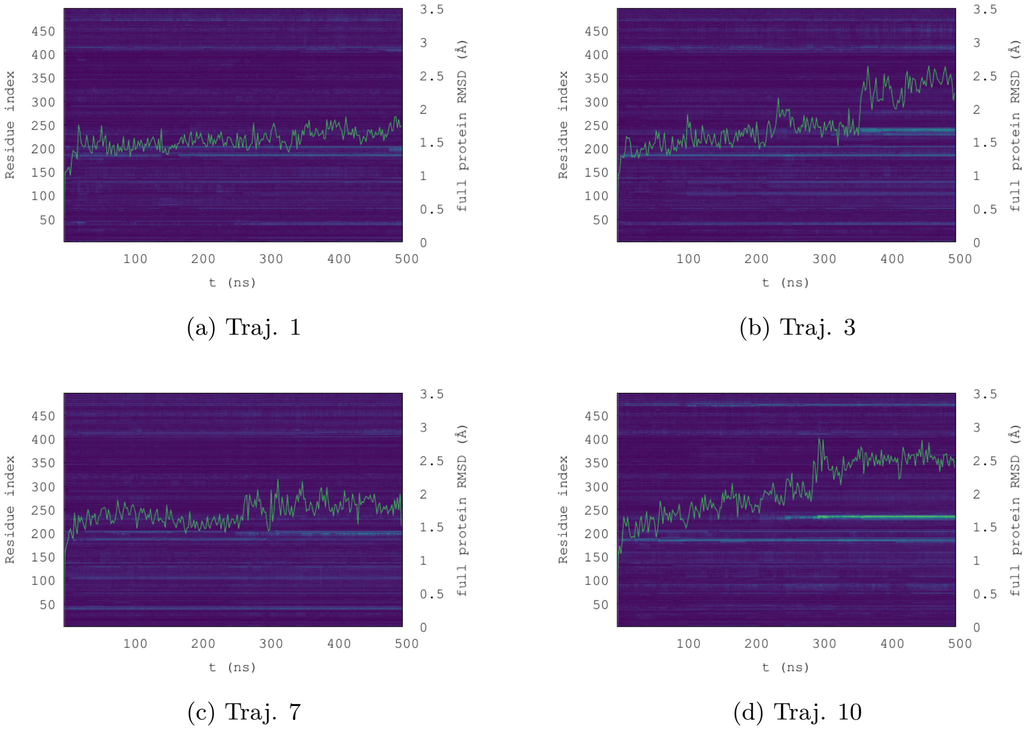

(a) Traj. 1

(b) Traj. 3

(c) Traj. 7

(d) Traj. 10

Figure S4: Per-residue RMSD of the backbone heavy atoms along four selected 500-ns MD trajectories of *Cl*Cry4 with 21 $O_2^{\bullet-}$ radicals. The average RMSD of the entire protein, as shown in Fig. S1 above, has been superimposed for comparison. All axes scaling and colour scales are harmonised, allowing the direct visual comparison across sub-figures. Based on synchronised full-protein and residue-resolved RMSD jumps, the marked RMSD jumps observed in Fig. S1 can here be identified as accompanying the release of the phosphate binding loop. This is particularly evident for simulations with Cl^-ions, in trajectories 3 (Fig. S5 (c)) and 4 (Fig. S5 (d)), and for trajectories 3 and 10 of the $O_2^{\bullet-}$simulations shown here. Other than this, areas of chronically elevated RMSD correspond to the high-RMSF peaks in Fig. S2, in particular the two peaks at residues 180-195 and 200-210. Interestingly, these seem to be also able to give rise to full-protein RMSD elevation ($O_2^{\bullet-}$: trajectory 7 from $\simeq 250\,$ns; Cl^-: trajectories 1 and 8 - see Fig. S5), although not nearly achieving the same magnitude as the phosphate binding loop.

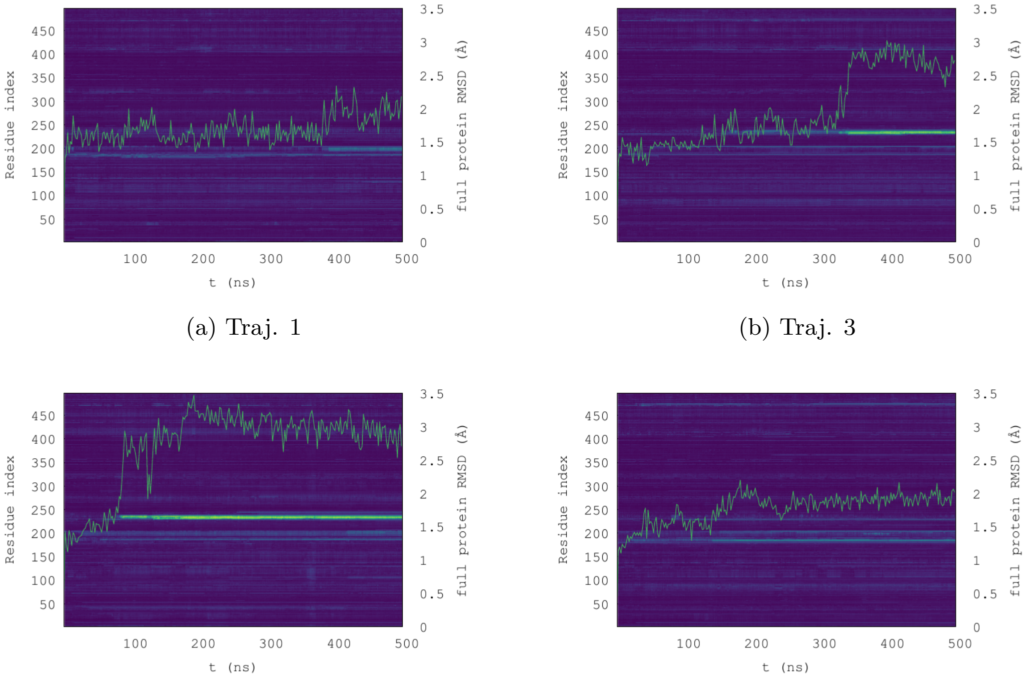

(a) Traj. 1 (b) Traj. 3

(c) Traj. 4 (d) Traj. 8

Figure S5: Per-residue RMSD of the backbone heavy atoms along four selected 500-ns MD trajectories of *Cl*Cry4 with 21 Cl^- counterions. See Fig. S4 for details.

ANION AFFINITY TO VARIOUS AMINOACID RESIDUES

Fig. S6 shows the probability of having a given aminoacid as the nearest neighbour to a Cl^- (in orange) and $O_2^{\bullet-}$ (in blue) ion, during a binding event. Chance encounters, *i.e.* very short encounters that do not trap the ion at the protein surface for an extended period of time, are excluded from the statistics by including only binding events extending over 2 or more consecutive frames. Since the time between 2 consecutive frames is 200 ps, this corresponds to a binding time of at least 200 ps.

$O_2^{\bullet-}$ shows well converged statistics, with only 44 % of its binding events being single-frame encounters, leaving 121, 235 frames of strong ion-protein interactions. $O_2^{\bullet-}$ shows an overwhelming affinity with arginine (Arg, $\simeq$ 40 %), followed by lysine (Lys, $\simeq$ 7 %) and leucine (Leu, $\simeq$ 5 %). The affinity with Arg and Lys is readily explained by the fact that

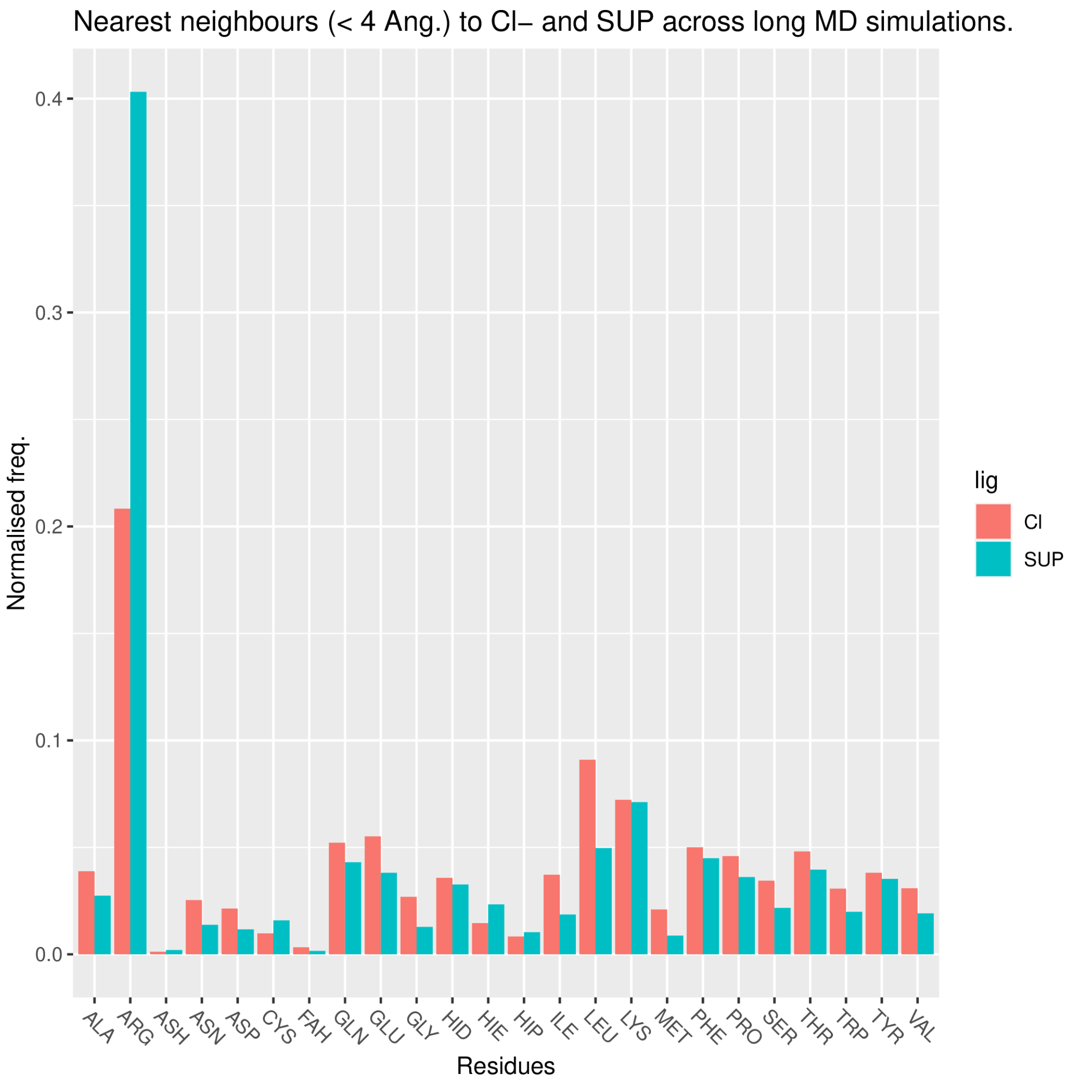

Figure S6: Probability of bound $O_2^{\bullet-}$ or Cl^- to have a specific aminoacid as its nearest neighbour. Binding is defined as the anion residing within 4 Å of the protein surface for at least 2 consecutive frames. Statistics have been accumulated over ten 500-ns MD simulations containing 21 anions for both scenarios.

these residues are positively charged at physiological pH, resulting in a potentially strong Coulombic attraction. Regarding the Leu residue, a neutral and apolar aminoacid, its relatively high rate of $O_2^{\bullet-}$ binding is unexpected. Upon visual inspection of the crypt of the protein, in particular in the areas of high $O_2^{\bullet-}$ affinity identified in Fig. 2, the

high apparent affinity appears to simply reflect the presence of Leu residues next to strongly binding residues. Indeed, Leu314 is located right next to Arg486, one of the key aminoacids for immobilising $O_2^{\bullet -}$; Leu354 is located between Arg409 and Arg419, two fairly flexible residues, which we have found to exchange $O_2^{\bullet -}$.

This binding statistics are less reliable for Cl^-, for which the vast majority (88 %) of binding events cover only one frame. The binding statistics are therefore only computed over 3879 frames for Cl^-, out of a total of $525,000$ ($2,500\,$frames $\times$ $10\,$trajectories $\times$ $21\,$ions). Cl^- too shows a marked preference for arginine ($\simeq 20\,\%$), followed by leucine ($\simeq 9\,\%$) and lysine ($\simeq 7\,\%$).

$O_2^{\bullet -}$ BINDING AT BINDING SITES

We explore the possibility that $O_2^{\bullet -}$ is simultaneously bound by multiple Arg residues, facilitating the transport from site-to-site, e.g. from site 1 to 2 via site 4. Figure S7 visualises the propensity of $O_2^{\bullet -}$ being close to specific Arg residues, specifically R365, R409, R415, and R419. Here, a characteristic numeric ID is associated to each combination of arginines binding the same $O_2^{\bullet -}$, attributed according to the following rule: R365 (near site 1) has ID = 1, R409 (site 4) has ID = 2, R415 (site 2) has ID = 4, and R419 (site 2) has ID = 8. If a $O_2^{\bullet -}$ is held by both R365 and R409 simultaneously, *i.e.* stays within 3.0 Å of both, this configuration is associated with ID = 1 + 2 = 3. The same logic applies to other multiple binding events. Figure S7 describes the binding interactions for one 500 ns-trajectory; it is representative of the whole set. A recurring feature is that the most common binding patterns are single-arginine binding, as shown by the nearly-solid lines of ID 2, 4, and 8. The only significant double-binding that occurs is at ID 12, *i.e.* R415 + R419, both located in site 2. In addition, ID 6 binding occurs (R409 + R415, *i.e.* cross-site binding), but is rare.

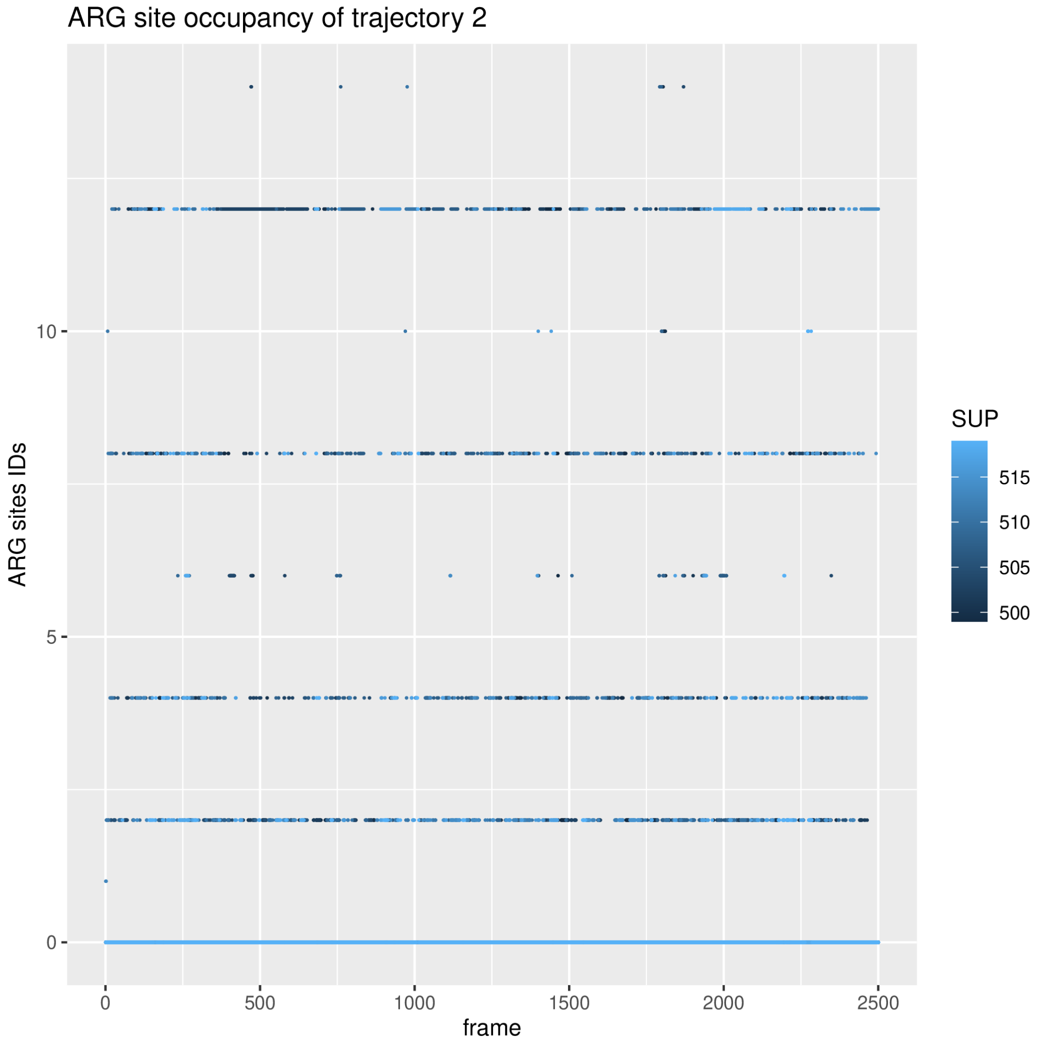

Figure S7: Occupancy of the various binding sites consisting of the possible combinations of R365, R409, R415, and R419. To each combination of binding arginines corresponds a unique ID, according to a naming protocol given in the main text. The colour code refers to the index of the $O_2^{\bullet -}$ anions in the system.

BINDING TIMES FROM BOUND FRACTIONS

Table S1: Results of fitting the time-dependence of the bound fraction, i.e. the fraction of initially bound configurations that are still bound at a particular time, using a bi-exponential function. The approach has been applied to the three different scenarios studied, namely I) the 10 500-ns trajectories of 21 freely-diffusing $O_2^{\bullet -}$; II) 440 restarted trajectories tracking the escape of a single $O_2^{\bullet -}$ from a binding site; and III) 100 trajectories of a single $O_2^{\bullet -}$ diffusing from a putative formation site near the flavin. The fitted model was of the form $p_1 \exp(-t/\tau_1) + (1 - p_1) \exp(-t/\tau_2)$, subject to $0 < p_1 \leq 1$. $\tau_{\text{eff}} = p_1\tau_1 + (1 - p_1)\tau_2$ is the effective binding time, R^2 the coefficient of determination and $\langle t_b \rangle$ the average binding time.

Condition	Site	τ_1 (ns)	τ_2 (ns)	p_1	R^2	τ_{eff} (ns)	$\langle t_b \rangle$ (ns)
I	all	0.93	15	0.988	0.949	1.10	1.13
	1	1.00	9	0.963	0.958	1.30	1.33
	2	1.10	8	0.955	0.967	1.43	1.43
	3	0.91	117	0.982	0.948	2.99	2.41
	4	0.80	–	1.	0.926	0.80	0.77
	5	0.78	–	1.	0.922	0.78	0.73
II	all	1.90	52	0.788	0.980	12.6	13.5
	1	2.06	9.1	0.740	0.998	3.9	3.8
	2	0.45	7.6	0.251	0.996	5.8	5.9
	3	4.95	75	0.377	0.997	48.7	49.1
	4	1.46	24	0.955	0.987	2.5	2.4
	5	0.43	1.4	1.000	0.985	0.4	0.4
III	all	2.98	32	0.944	0.995	4.6	4.7
	1	2.72	48	0.893	0.965	7.5	7.0
	2	3.75	52	0.937	0.996	6.8	6.9
	4	3.37	75	0.981	0.996	4.7	4.5
	5	1.67	6.5	0.72	0.991	3.0	3.0

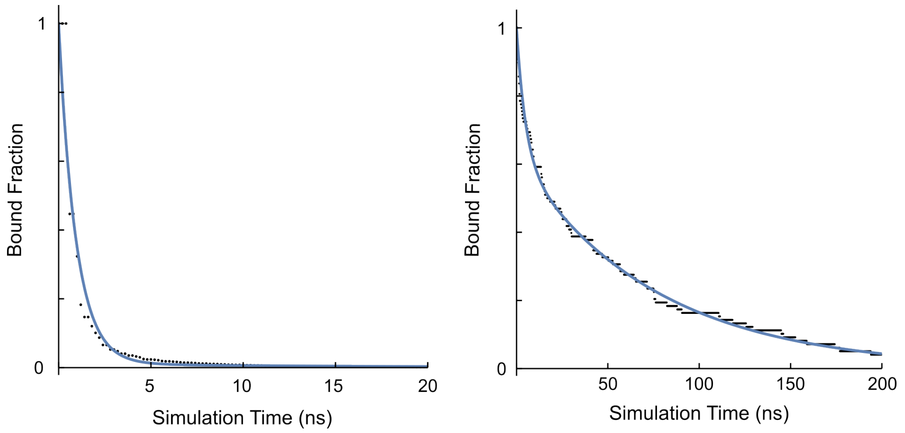

Figure S8: Fraction of bound superoxide molecules versus the simulated time for superoxide bound at any site as sampled for condition I (left) and for superoxide escaping from site 3 (right; condition II, in Tab. S1). Simulation data are represented as symbols, and bi-exponential fits of the data are represented by solid lines.

$O_2^{\bullet -}$ BOUND AT AND ESCAPING FROM BINDING SITES

We report binding times, t_b, and rotational correlation times, τ_2, for $O_2^{\bullet -}$ trapped in one of the binding sites identified in the main text. As above, bound configurations are defined as those with a protein-$O_2^{\bullet -}$ distance of less than 3.0 Å. Tables S2 through S6 collect average t_b and τ_2 values computed for the 5 sets of 20 replica trajectories associated with each site (2 in the case of site 4 - see main text). Data are reported individually for every starting configurations due to sometimes large intra-site variability. Note further that averages were not computed for the full set of 20 replica trajectories, but only for trajectories for which a τ_2 characteristic of a bound configuration, *i.e.* $\tau_2 \geq 10.0$ ps, was determined. For each set, the actual number of trajectories on which the calculated $\langle \tau_2 \rangle$ and $\langle t_b \rangle$ are based is reported as N. "N/A" is reported in the case that none of the trajectories gave rise to $\tau_2 \geq 10.0$ ps, or as in the case of site 5 frame 1, to a calculable τ_2 at all. This can occur when the bound configurations are too short-lived (here: $\langle t_b \rangle = 0.11$ ns, with $t_b^{\max} = 0.26$ ns) to evaluate the autocorrelation function $C_2(t)$. In order to evaluate correlation times, we fitted either the first quarter of the

Table S2: Average binding times and rotational correlation times τ_2 of $O_2^{\bullet -}$ in site 1, calculated from 20 MD trajectories for 5 initial binding configurations each, i.e. a total of 100 trajectories. 69 trajectories yielded $\tau_2 \geq 10.0$ ps and were included in the statistics. Standard deviations are reported as a mean to describe the spread of values observed.

		Occupancy time (ns)	τ_2 (ps)	N
	1	7.07 ± 9.18	108.8 ± 180.4	16
	2	1.26 ± 0.99	17.9 ± 7.4	5
site 1	3	3.69 ± 3.02	99.9 ± 136.9	8
	4	3.92 ± 4.17	66.4 ± 52.4	12
	5	7.30 ± 6.46	103.6 ± 101.3	17

Table S3: Average binding times and rotational correlation times τ_2 of $O_2^{\bullet -}$ in site 2, calculated from 20 MD trajectories for 5 initial binding configurations each. 66 trajectories yielded $\tau_2 \geq 10.0$ ps and were included in the statistics.

		Occupancy time (ns)	τ_2 (ps)	N
	1	2.10 ± 1.69	18.2 ± 3.5	4
	2	6.30 ± 3.90	97.2 ± 65.8	18
site 2	3	9.30 ± 9.62	92.5 ± 107.6	18
	4	10.87 ± 10.71	120.6 ± 212.7	17
	5	6.89 ± 5.32	56.9 ± 51.7	9

ACF, or the part up to the point where it reaches 0 for the first time – whichever came first (typically the latter).

The degree of immobilisation imposed by the binding residues in binding sites can be appreciated by comparing with τ_2 values evaluated for freely-tumbling, bulk $O_2^{\bullet -}$ radicals. Such trajectories were generated in a separate MD simulation, where 5 $O_2^{\bullet -}$ radicals were left to tumble in a water box for 5 ns. Due to the expected fast rotational

Table S4: Average binding times and rotational correlation times τ_2 of $O_2^{\bullet-}$ in site 3, calculated from 20 MD trajectories for 5 initial binding configurations each. 78 trajectories yielded $\tau_2 \geq 10.0$ ps and were included in the statistics.

		Occupancy time (ns)	τ_2 (ps)	N
	1	10.11 ± 17.11	36.7 ± 32.8	12
	2	6.86 ± 6.30	59.8 ± 57.3	17
site 3	3	119.96 ± 113.23	116.2 ± 147.1	15
	4	65.76 ± 50.00	78.0 ± 108.3	19
	5	75.44 ± 71.02	59.8 ± 78.0	15

Table S5: Average binding times and rotational correlation times τ_2 of $O_2^{\bullet-}$ in site 4, calculated from 20 MD trajectories for 2 initial binding configurations each. 18 trajectories yielded $\tau_2 \geq 10.0$ ps and were included in the statistics.

		Occupancy time (ns)	τ_2 (ps)	N
site 4	1	0.65 ± 0.00	27.8 ± 0.0	1
	2	3.96 ± 8.11	61.6 ± 63.5	17

Table S6: Average binding times and rotational correlation times τ_2 of $O_2^{\bullet-}$ in site 5, calculated from 20 MD trajectories for 5 initial binding configurations each. 9 trajectories yielded $\tau_2 \geq 10.0$ ps and were included in the statistics.

		Occupancy time (ns)	τ_2 (ps)	N
	1	N/A	N/A	0
	2	0.52 ± 0.29	18.1 ± 9.0	4
site 5	3	0.59 ± 0.30	12.3 ± 0.5	2
	4	1.01 ± 0.13	15.0 ± 1.1	2
	5	0.69 ± 0.00	10.9 ± 0.0	1

dynamics, the time step between two consecutive saved geometries was reduced to 100 fs. Bulk $O_2^{\bullet -}$ achieved an average τ_2 of $\langle \tau_2 \rangle = 0.99 \pm 0.33$ ps.

BINDING ENERGIES OF ANIONS TO ARGININE: FORCE-FIELD VS. DFT

The intermolecular non-bonded interaction energy of arginine (Arg; labelled A) and the anions $O_2^{\bullet -}$ and Cl^- (labelled B) were calculated based on

$$E_{\text{int}}^{\text{AB}} = E^{\text{AB}} - \left(E^{\text{A}} + E^{\text{B}}\right), \tag{S1}$$

where E^{AB} is the potential energy of the complex of molecules A and B, and E^{A} and E^{B} are the potential energies of molecule A alone and molecule B alone, respectively, in the same geometry as in the complex. We compared interaction energies derived from MM (using standardised parameters for Cl^- and arginine and a symmetrically distributed charge of -0.5 for each O-atom in $O_2^{\bullet -}$in combination with the Lennard-Jones parameters as provided by the GAFF2 force field for oxygen) and DFT (on the CAM-B3LYP/def2-TZVP level of theory with D3BJ dispersion correction in Gaussian 16).

First, the interaction energy was calculated for complexes of $O_2^{\bullet -}$ and arginine, as this is a recurring interaction pattern for which MD-generated geometries are abundant, and one which is of particular significance for superoxide immobilisation. 36 consecutive conformations of a Arg-$O_2^{\bullet -}$ complex were extracted as a subtrajectory of one of the 500-ns trajectories described above and used to evaluate E^{AB}. Since the phenomenon under investigation here is long, uninterrupted immobilisation of $O_2^{\bullet -}$ by the protein, it seemed that more relevant energetic figures would be obtained by treating a subtrajectory corresponding to a bound state rather than arbitrary snapshots. The arginine residue was truncated at the backbone, leaving an unsaturated valence at the amino N and carboxylate O, which was capped at by a methyle and an acetyl group, respectively. This method avoided creating a non-standard residue for arginine. A short geometry optimisation in MM was performed for these structures, whereby the arginine side-chain, its backbone and the anion were kept fixed using a strong harmonic potential, while the

Table S7: Mean interaction energies and standard deviations of 36 samples of Arg-$O_2^{\bullet-}$-complexes and derived Arg-Cl^--complexes, computed at the MM and QM (DFT) level.

Complex	$E_{\text{int}}^{\text{MM}}$ (kcal/mol)	$E_{\text{int}}^{\text{QM}}$ (kcal/mol)
$[O_2^{\bullet-} - \text{ARG}]$	-92.0 ± 3.6	-113.0 ± 3.9
$[Cl^-_{\text{opt}} - \text{ARG}]$	-86.1 ± 1.8	-109.0 ± 1.9
$[O^{\bullet-}_{2,\text{opt}} - \text{ARG}]$	-99.6 ± 2.4	-118.0 ± 1.9

capping residues were allowed to relax. As for Cl^-extended binding events were much rarer and for better comparability with the Arg-$O_2^{\bullet-}$ complexes, the Arg-Cl^- complex geometries were obtained from the $O_2^{\bullet-}$-subtrajectory by mutating the $O_2^{\bullet-}$ to Cl^-. Both the anion and the capping residues were allowed to relax in the subsequent MM energy minimisation. This protocol permit to approximate a more realistic binding configuration for Cl^- ions. For comparison, for the $O_2^{\bullet-}$trajectories too, a third set of geometries was constructed for which the anion was allowed to relax. The three sets of ion-arginine binding geometries thus created will be referred to as $[O_2^{\bullet-} - \text{ARG}]$, $[Cl^-_{\text{opt}} - \text{ARG}]$, and $\left[O^{\bullet-}_{2,\text{opt}} - \text{ARG}\right]$ in the sequel. The "opt" subscript in the latter sets recalls the fact that the anion was allowed to relax its position with respect to the arginine, therefore realising an idealised binding configuration. Finally, interaction energies were computed on these sets of geometries on the level of MM and DFT. The Basis Set Superposition Error (BSSE) in DFT, was compensated using the Counterpoise correction in Gaussian 16 [2, 3]. For the clusters studied here, the BSSE amounted to $\simeq 4$ kcal/mol, a chemically significant error if left uncorrected. Table S7 summarises our results.

For both anions, the force-field underestimates E_{int} by about 20% over the DFT energies; specifically, -21.2 kcal/mol *i.e.* 19% for $O_2^{\bullet-}$, and -22.8 kcal/mol *i.e.* 21% for Cl^-. Crucially, $O_2^{\bullet-}$ interaction energies are not overestimated by the force-field, meaning that the length and quality of immobilisation of binding events observed in the dynamics constitutes a "worst-case scenario". While it may seem surprising that

for Cl^-, a common biological ion for which force-field parameters were derived with great care, the binding energy is not exactly reproduced in MM, one must keep in mind that forcefield parameters are not fitted to reproduce ion-residue interaction energies, but rather “solvation free energies, radial distribution functions, ion-water interaction energies and crystal lattice energies and lattice constants” (cited from the AMBER website) [4].

If the $O_2^{\bullet -}$ was also left to relax its position with respect to the arginine, realising configuration $\left[O_{2,\mathrm{opt}}^{\bullet -} - \mathrm{ARG}\right]$, the binding was further strengthened, yielding MM interaction energies of $E_{\mathrm{int}}^{MM} = -99.6 \pm 2.4\,\mathrm{kcal/mol}$. Besides the averaged picture, each of the $\left[O_{2,\mathrm{opt}}^{\bullet -} - \mathrm{ARG}\right]$ structures registered an decrease in E_{int} after letting $O_2^{\bullet -}$ relax. This demonstrates that structural relaxation boosts the interaction energy. We can therefore make the proposition that Cl^- interacts in a significantly weaker fashion with the arginine residue than $O_2^{\bullet -}$. Obviously, as the subtrajectory chosen might not be representative of the broad range of binding geometries accessible to both ions, which often involve multiple residues, care needs to be exercised in extrapolating from these findings to protein-anion binding in general. In any case, the data provide a validation of the approach and tentative explanation in terms of intrinsically stronger binding of $O_2^{\bullet -}$.

ENERGETICS OF DESOLVATION: $O_2^{\bullet -}$ VS. CL^-

Figure 7 in the main text reports the radial distribution functions (RDFs) of water with respect to $O_2^{\bullet -}$ and Cl^- for the ions in the *bound* and *bulk* state. The RDFs were computed over the ten 500-ns MD simulations containing, respectively, 21 $O_2^{\bullet -}$ and 21 Cl^- ions. A $O_2^{\bullet -}$ radical was considered to be in a *bound* state when found within 2.75 Å of the protein surface, and a Cl^- ion within 3.00 Å; the *bulk* state was thought to be realised for distances larger than 10.0 Å from the protein. In general, the RDF between

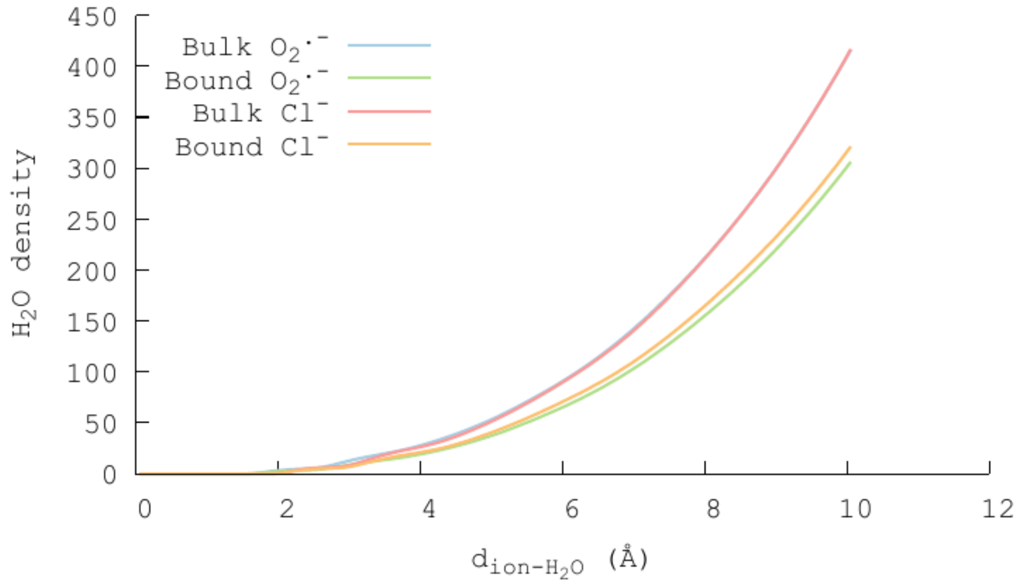

Figure S9: Number of water molecules in the solvation shell of $O_2^{\bullet -}$ and Cl^- ions as provided by the integrated Radial Distribution Functions (RDF) of the ions with respect to H_2O, when in a bound or bulk configuration. The RDFs were computed over all frames of the ten 500-ns MD simulations, and all 21 ions, which satisfied the distance criteria.

a molecule i and the water molecules w, $g_{iw}(r)$, is given by

$$g_{iw}(r) = \frac{1}{4\pi r^2 N_w \Delta r} \sum_{t=0}^{T} \sum_{j=1}^{N_w} \delta \left[|r_i(\vec{t}) - r_j(\vec{t})| - r \right], \tag{S2}$$

where T is the simulation length, N_w the total number of water molecules, and $\frac{1}{4\pi r^2 \Delta r}$ the normalisation volume on a grid of spacing Δr [5]. The RDF is a measure of the angle-averaged density of w with respect to i at distance r, and is here used to uncover solvation shells. Integrating the g_{iw} functions yields the number of water molecules as a function of distance from molecule i, and therefore can be used to count the number of water molecules in a given solvation shell. The integrated RDFs are shown in Figure S9, and a numerical breakdown is given in Table 4 in the main text.

The free energy of desolvation can be computed as the energy difference between

the solvated state and the molecule(s) in the gas-phase, *i.e.* in vacuum [6]. Here, we explicitly account for the water molecules of the first solvation shell. The solvation of representative water-ion complexes was then modelled using DFT in combination with an implicit solvation model–the polarisable continuum model (PCM). The advantage of using a PCM instead of an explicit solvation, where water molecules are provided in a given geometry and are explicitly part of the DFT calculation, is the built-in treatment of entropic effects: indeed, a PCM approximates the average solvation pattern around the solute, by effectively modelling all possible solvent arrangements. The free energy of desolvation, ΔG_{desolv}, was thus calculated for $O_2^{\bullet -}$ and Cl^- using

$$\Delta G_{\text{desolv}} = E^{\text{gas}} - E^{\text{PCM}} + C, \tag{S3}$$

where E^{gas} and E^{PCM} are respectively the energy of the ion-water system including the first solvation shell in the gas-phase and with PCM solvation. $C = -RT \ln(55.5)$, with 55.5 M the molarity of water. Energies were computed using DFT as implemented in Gaussian 16, at the CAM-B3LYP/def2-TZVPP level of theory with D3BJ empirical dispersion. Cavities for the PCM models were defined with the Unified Atom model calibrated based on Kohn-Sham DFT calculations and the Integral Equations Formalism (IEF-PCM) was used.

Since both anions do not shed the entirety of their solvation shell when entering a bound state, the desolvation energy associated with binding was calculated for explicit complexes representing the first solvation shell in the bound, partial solvated state, and its free state. To this end, we built an explicit solvation shell around the anions, assuming an octohedral coordination geometry for simplicity. H_2O molecules were placed at a distance informed by the RDFs shown in Fig. 7 in the main text. Their relative orientation to the anion, shown in Figure S10 (a), with the hydrogen pointing at the anion, was informed both by chemical intuition − electrostatic interactions favour this solvation pattern, with H atoms with a positive partial charge neutralising the anion − and by the $g_{\text{ion}-\text{O}}$ and $g_{\text{ion}-\text{H}}$ functions. The $g_{\text{ion}-\text{H}}$ function shows a density peak at a shorter distance than $g_{\text{ion}-\text{O}}$, indicating that the H_2O H atoms are pointing towards the anions. The solvation shell was built for both $O_2^{\bullet -}$ and Cl^- anions in a stepwise

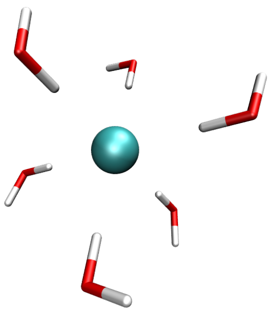

(a) 1st H_2O shell around Cl^- ion

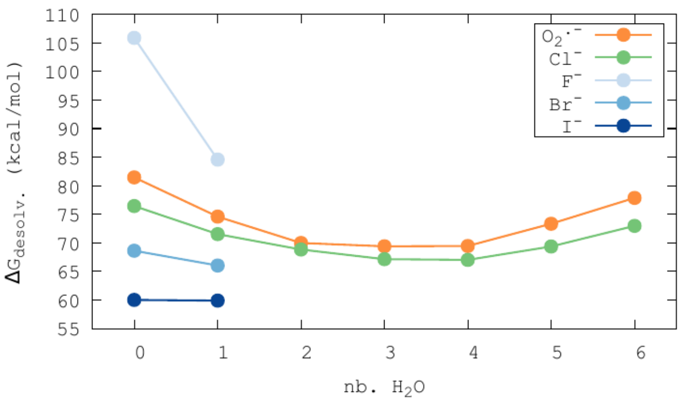

(b) $\Delta G_{\text{desolv.}}$ of various hydrated ions

Figure S10: (a) Model of a hexa-hydrated Cl^- ion. (b) Free energies of desolvation of the N-hydrated ion X (X = $O_2^{\bullet-}$, Cl^-, F^-, Br^-, I^-), computed using DFT at the CAM-B3LYP/def2-TZVPP level in `Gaussian 16`, with and without PCM implicit solvation.

manner, adding one H_2O molecule at a time and computing $\Delta G_{\text{desolv.}}$ on this partial solvation shell geometry. This process was also partially repeated for other halide anions not present in the system. Our results are reported in Figure S10 (b).

The solvation free energies of isolated ions were well reproduced, registering a deviation of about 5 kcal/mol with respect to reference values derived from experimental data [7]. Using data reported in Table 4, the desolvation free energy of a $O_2^{\bullet-}$ solvated by 3.63 water molecules, *i.e.* its bound-state hydration shell, is $\Delta G_{\text{desolv.}}^{O_2^{\bullet-}} \simeq 70\,\text{kcal.mol}^{-1}$; and for a Cl^- surrounded by 5.61 H_2O, $\Delta G_{\text{desolv.}}^{Cl^-} \simeq 72\,\text{kcal.mol}^{-1}$. Their very similar desolvation energies do not permit to rationalise the large discrepancy in binding abilities apparent in this study. Obviously, the estimation made here is not quantitative, chiefly due to the absence of entropic effects in the first solvation shell and due to its arbitrary conformation. Nevertheless, the very similar desolvation energies of $O_2^{\bullet-}$ and Cl^- ions, all other parameters being equal, is an indicator that desolvation is not a strong driver, if at all, in the binding capabilities of the two anions to the protein.

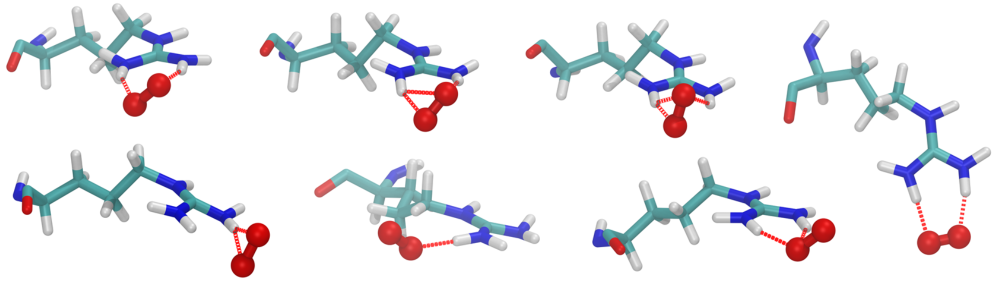

Figure S11: Small selection of configurations observed for superoxide bound to an arginine residue involving one to three simultaneous hydrogen bonds with hydrogen atoms from one or both of the ε-N atoms of the arginine guanidino-group.

CORRELATION OF BINDING WITH H-BONDING

In this section, we seek to clarify the relationship between t_b and τ_2, as well as explore the possibility of the involvement of hydrogen bonding for immobilising $O_2^{\bullet -}$. To this end, we have monitored the rate of occurrence of hydrogen bonding, between a $O_2^{\bullet -}$ and the amino acids composing its trapping site. H-bonding was assumed for a donor-acceptor atom distance of $d_{\mathrm{DA}} \leq 3.0\,\text{Å}$ and a binding angle $\widehat{\mathrm{DHA}} \leq 150\,°$. Figure S11 illustrates typical configurations from the varied ensemble of configurations of superoxide hydrogen-bonded to arginine. Figure S12 reports τ_2 and t_b in combination with the fractional occurrence of a certain type of hydrogen bonding along each trajectory. In particular, we look at the proportion of "double" H-bonding, *i.e.* the fraction of the trajectory where both $O_2^{\bullet -}$ atoms are simultaneously involved in a H-bond; and the "no-" H-bond case, where none of them is involved. Only trajectories involving stable binding with $t_b \geq 10\,\mathrm{ns}$ have been considered here.

There is no evidence of a linear correlation between τ_2 and t_b (Pearson correlation coefficient: 0.10). This goes against the intuitive reasoning that long $O_2^{\bullet -}$ trapping times and strong immobilisation are associated. This representation almost shows the opposite trend: long t_b overwhelmingly have associated $\tau_2 \leq 100\,\mathrm{ps}$, and the 2 longest τ_2 both

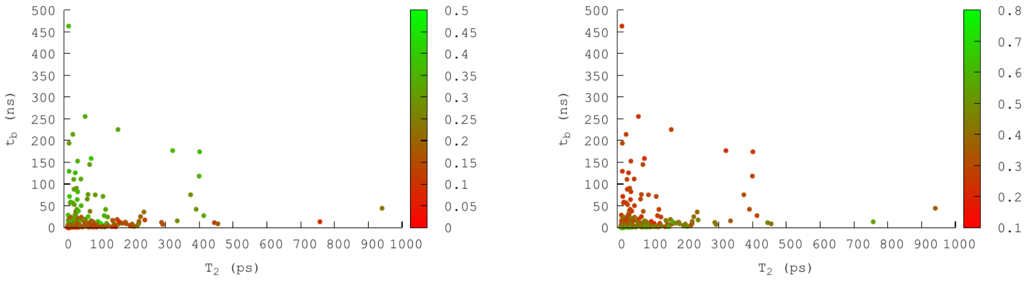

(a) **Double** H-bonding (b) **No** H-bonding

Figure S12: t_b vs. τ_2 of $O_2^{\bullet-}$ bound at/escaping from binding sites with the probability of (a) double H-bonding and (b) absence of H-bonding throughout the simulation encoded in the colour of markers. Only trajectories with $t_b \geq 10$ ns have been considered.

have $t_b \leq 50$ ns. The nearly vertical arrangement of points near $\tau_2 = 400$ ps illustrates the lack of correlation between these two properties: a very large τ_2 can be achieved with $O_2^{\bullet-}$ binding times anywhere from 10 to 200 ns.

Since length of binding is not a predictor of the quality of immobilisation, we focus on H-bonding interactions. The colour scales in Fig. S12 indicate the proportion of (a) double H-bonding, and (b) the absence of H-bonding. No correlation can be detected upon visual inspection, for either double- or non-bonding. This is confirmed by the Pearson correlation coefficients, which are $r_{\tau_2/2Hb} = 0.06$ for τ_2 correlation with double-bonding and $r_{\tau_2/0Hb} = -0.10$ for the absence of H-bonding. Again, this is rather surprising: intuitively, one would expect that simultaneous binding of both atoms of $O_2^{\bullet-}$ would prevent it from reorienting. Here, only the signs of the linear correlation coefficients confirm this hypothesis; but while they hint at a positive correlation of τ_2 with double H-bonding, and an inverse correlation with the absence of H-bonding, their magnitude is too small to assign the variance in τ_2 to either of these properties. The lack of correlation between τ_2 and the simultaneous binding of both $O_2^{\bullet-}$ atoms is a surprising finding, which warrants further investigation into the subject. Perhaps this could be traced to the great flexibility shown by arginine residues, which do not maintain a persistent H-bonding

scaffolding to anchor the $O_2^{\bullet-}$ radical.

There is, on the other hand, a clearer correlation between t_b and the degree of hydrogen bonding in which $O_2^{\bullet-}$ is involved while in the binding site. Upon visual inspection of Fig. S12, is seems that a larger proportion of double H-bonding correlates with larger t_b, while a larger fraction of no H-bonding at all correlates with short t_b. This observation is confirmed by the Pearson correlation coefficients, which are $r_{t_b/2Hb} = 0.48$ with double-bonding and $r_{t_b/0Hb} = -0.44$. This moderately strong linear correlation makes intuitive sense, as one can expect that more numerous interactions between $O_2^{\bullet-}$ and the site stabilise the bound configuration and therefore increase the trapping time. The representation in Fig. S12 only includes trajectories for which $\tau_2 \geq 10.0\,\text{ps}$. Extending this to the full set does not change the conclusion: the Pearson correlation coefficient of t_b with double H-bonding is 0.49, and remains at -0.44 with no-H-bonding.

A TOY ELECTROSTATICS MODEL

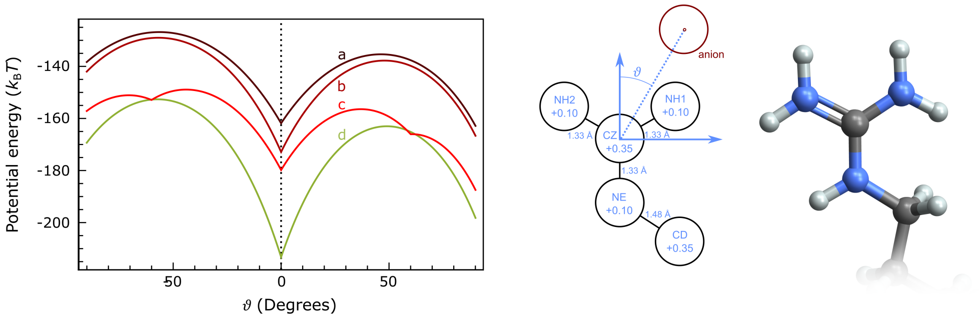

Figure S13: Toy electrostatic model of the interaction energy of chloride and superoxide anions with the arginine sidechain. The sidechain was abstracted in term of the heavy atoms composing the guanidino group with charges assigned based on the PARSE force field (see the middle figure for an illustration of the underlying structural abstraction and assigned charges). The structure has been assumed planar, the guanidino-bond angles 120 degrees, and the bond angle involving the terminal CD-atom 123 degrees. The superoxide bond length has been chosen as 1.31 Å; all other bond length are reported in the figure. An atomistic model of the sidechain is shown on the right. The electrostatic potential energy, reported in the left graph, has been calculated in vacuum as a function of the angle, ϑ, subtended by the anion centre of mass and the NE-CZ-direction (see schematic in the middle) for the following scenarios: a) the Cl^- anion placed in the plane of the guanidino group such that the distance to the closest heavy atom of the group amounted to 2.4 Å (maximum of RDF), b) the superoxide anion in the guanidino plane and aligned with the ray at angle ϑ such that the minimal distance amounted to 1.8 Å, c) the superoxide anion such that the anion was oriented perpendicular to the ϑ-ray within the guanidino plane at a minimal distance of 1.8 Å and d) the superoxide anion such that the oxygen atoms where oriented perpendicular to the guanidino plane at a distance of half the bond length such that the minimal distance amounted to 1.8 Å.

* d.r.kattnig@exeter.ac.uk

[1] F. Schuhmann, D. R. Kattnig, and I. A. Solov'yov, Exploring post-activation conformational changes in pigeon cryptochrome 4, J. Phys. Chem. B **125**, 9652 (2021).

[2] S. F. Boys and F. Bernardi, The calculation of small molecular interactions by the differences of separate total energies. some procedures with reduced errors, Molecular Physics **19**, 10.1080/00268977000101561 (1970).

[3] S. Simon, M. Duran, and J. J. Dannenberg, How does basis set superposition error change the potential surfaces for hydrogen-bonded dimers?, Journal of Chemical Physics **105**, 10.1063/1.472902 (1996).

[4] I. S. Joung and T. E. Cheatham, Determination of alkali and halide monovalent ion parameters for use in explicitly solvated biomolecular simulations, Journal of Physical Chemistry B **112**, 10.1021/jp8001614 (2008).

[5] V. Lounnas, B. E. Pettitt, and G. N. Phillips, A global model of the protein-solvent interface, Biophysical Journal **66**, 10.1016/S0006-3495(94)80835-5 (1994).

[6] A. Brugnara, F. Topić, K. Rissanen, A. D. L. Lande, B. Colasson, and O. Reinaud, Selective recognition of fluoride anion in water by a copper(ii) center embedded in a hydrophobic cavity, Chemical Science **5**, 10.1039/c4sc01457j (2014).

[7] M. D. Tissandier, K. A. Cowen, W. Y. Feng, E. Gundlach, M. H. Cohen, A. D. Earhart, J. V. Coe, and T. R. Tuttle, The proton's absolute aqueous enthalpy and gibbs free energy of solvation from cluster-ion solvation data, Journal of Physical Chemistry A **102**, 10.1021/jp982638r (1998).
